# Investigation of electrocatalytic and photocatalytic ability of Cu/Ni/TiO_2_/MWCNTs Nanocomposites for detection and degradation of antibiotic drug Furaltadone

**DOI:** 10.1038/s41598-022-04890-z

**Published:** 2022-01-18

**Authors:** Dhanapal Vasu, Arjunan Karthi Keyan, Subramanian Sakthinathan, Te-Wei Chiu

**Affiliations:** Department of Materials and Mineral Resources Engineering, National Taipei University of Technology, No. 1, Section 3, Chung-Hsiao East Road, Taipei 106, Taipei, Taiwan, ROC

**Keywords:** Materials science, Nanoparticles, Nanowires, Pollution remediation

## Abstract

In this manuscript, “Get two mangoes with one stone” strategy was used to study the electrochemical detection and photocatalytic mineralization of furaltadone (FLT) drug using Cu/Ni/TiO_2_/MWCNTs nanocomposites for the first time. The bi-functional nanocomposites were synthesized through a hydrothermal synthesis technique. The successfully synthesized nanocomposites were analyzed by various analytical techniques. The Cu/Ni/TiO_2_/MWCNTs nanocomposites decorated screen-printed carbon electrode (SPCE) exhibit a good electrocatalytic ability towards detection of FLT. Moreover, the electrocatalytic detection of FLT based on the nanocomposites decorated SPCE have high stability, lower detection limit, and excellent sensitivity of 0.0949 μM and 1.9288 μA μM^−1^ cm^−2^, respectively. In addition, the nanocomposites decorated SPCE electrodes performed in real samples, such as river water and tap water, the satisfactory results were observed. As UV–Visible spectroscopy revealed that the Cu/Ni/TiO_2_/MWCNTs nanocomposites had an excellent photocatalytic ability for degradation of FLT drug. The higher degradation efficiency of 75% was achieved within 45 min under irradiation of visible light. In addition, after the degradation process various intermediates are produced which is confirmed by GC–MS analysis. The excellent photocatalytic ability was improved to the dopant ions and restrictions of electron–hole pair.

## Introduction

Furaltadone (FLT) is an antibiotic drug and family of nitrofuran group, which is generally used as a feed additive for growth evolution and, to prevent protozoan and bacterial infections in livestock, bee colonies, and aquaculture^1–4^. Furaltadone is an antibiotic agent and a synthetic chemotherapeutic agent, which is present in animal foodstuffs such as cattle, poultry, fish, and shrimp^5–7^. However, FLT and its metabolites exhibit significant carcinogenic and mutagenic characteristics for all living organisms. Hence, the FLT usage of animal-borne food production has already been banned by the European Union (EU) since the mid-1990s, which the usage level in aquaculture products was set at 1 μg/kg^1,8,9^. Furthermore, the usage of FLT-related antibiotics in animal-borne food manufacture has also been banned in a lot of countries such as Australia (1993), the United States (2000), the Philippines (2001), China (2002), Brazil (2002), and Thailand (2002)^10,11^. However, FLT or nitrofurans-related antibiotics are still generally used in many countries around the world, while still exist metabolites in other countries especially developing countries. If FLT is entered into the human body it can be rapidly metabolized as it is more sensitive to light^1,12^.

In this regard, the identification and removal of FLT are more essential and needed much. Therefore, to identify the quantity of FLT and its metabolites in imported and exported foods. There are numerous methods are available for the detection of FLT antibiotic drugs such as ELISA, LC–MS, HPLC, Spectrophotometry, fluorimetry, and LC–MS/MS. However, those analytical methods have some limitations for FLT detection like longer duration of results, high costs, and not being easy to handle^12,13^. To overcome those limitations, electrochemical techniques are the most preferred technique because its rapid response, low cost, simplicity, reliability, excellent sensitivity, and excellent selectivity. Moreover, the advantages of the electrochemical method are the best technique for analyzing real samples compared to other techniques.

Furthermore, the continuous release of FLT into the green environment to improper disposal from hospitals or industries causes chronic toxicity in all living organisms. Therefore, the mineralization of FLT from the industries is a major concern for many researchers and industrialists^14^. There are lots of traditional methods are available for the removal of FLT from the wastewater such as sonolysis, adsorption, coagulation, and ozonation. Among these methods, photocatalysis has attracted more attention due to its lost cost method and powerful technique as well as being eco-friendly. Fujishima and Honda have established photoelectrochemical cells (PEC) through the flowing of excited electrons from the harvesting of solar energy^15^. Many researchers carried out to attain photoanode, having a special characteristic of efficient visible lights absorption, proper bandgap energy, high photoconversion efficiency, low cost, and durability in the ideal PEC cell. The transition metal oxide materials exhibit sustainable performance in the PEC cell photoelectrode such as TiO_2_, ZnO, WO_3,_ and BaTiO_3_. Among those oxide materials, TiO_2_ has a more attractive n-type semiconductor, which has been widely used as a photo-electro catalyst due to its easily available or accessible, environmentally free, and high chemical stability. Nonetheless, it has some drawbacks such as large bandgap (3.2 eV), active under UV region only, low efficiency, and fast recombination of photo-generated electron–hole pair^16–18^. In addition, it has some electrical properties limitations such as less ionic diffusivity, poor conductivity in electrodes, and an electrode/electrolyte solution it has high resistance^19^.

To address the limitations, an important strategy has been implemented to enhance the electrochemical properties. Moreover, TiO_2_ has a surface oxygen defect within its crystal because it is a self-doped n-type material. Hence, the oxygen defect influences the TiO_2_ physio-chemical properties, whereas it will increase the light absorption capacity due to decreasing the bandgap. The oxygen defect serves as an electron donor, improved the electrical properties, which thereby strongly influenced the photo-electrochemical properties of TiO_2_^20,21^. A lot of techniques have been conducted to minimize the drawbacks and to enhance the photoconversion efficiency of TiO_2_ including spectral sensitization, controlling the valence band with help of orbitals **s** of **p** block metal ions and **p** of anions^17^. Metal ions doping into TiO_2_ has been a simple technique, cost-effective and repeatable synthesis pathway^22^. If doping of 3d transition metals into TiO_2_ such as Cu, Cr, Ni, Fe, V, and Mn has been enhancing the light absorbance capability to the visible region via the formation of mid-gap states^17,23–25^. Past few years, many scientific researchers have studied that TiO_2_ doping with transition metals like Au and Cu, Ni and Cu, Ni and N, and Fe and Ni can improve the photocatalytic activity than undoped TiO_2_^5,26^. Among these, Cu and Ni can be considered as the most favorable dopants for TiO_2_ in PEC cells due to the copper oxides (Cu^2+^ and Cu^+^) enhanced photocatalytic activity than the other metals because of its narrow bandgap (1.4 and 2.2 eV), high light absorption coefficient, and negative conduction band position (− 0.96 and − 0.22 V)^27^. Concurrently, Ni as a high-cost transition metal has been suggested for enhancing the photocatalytic ability for semiconductors to enhancing the thermal stability and controlling the surface morphology of photocatalysis^17,28,29^. Bashiri et al. investigated that the Cu/Ni doped TiO_2_ has lowest bandgap and absorb more light in the visible region from 400 to 800 nm.

**Figure 1:**
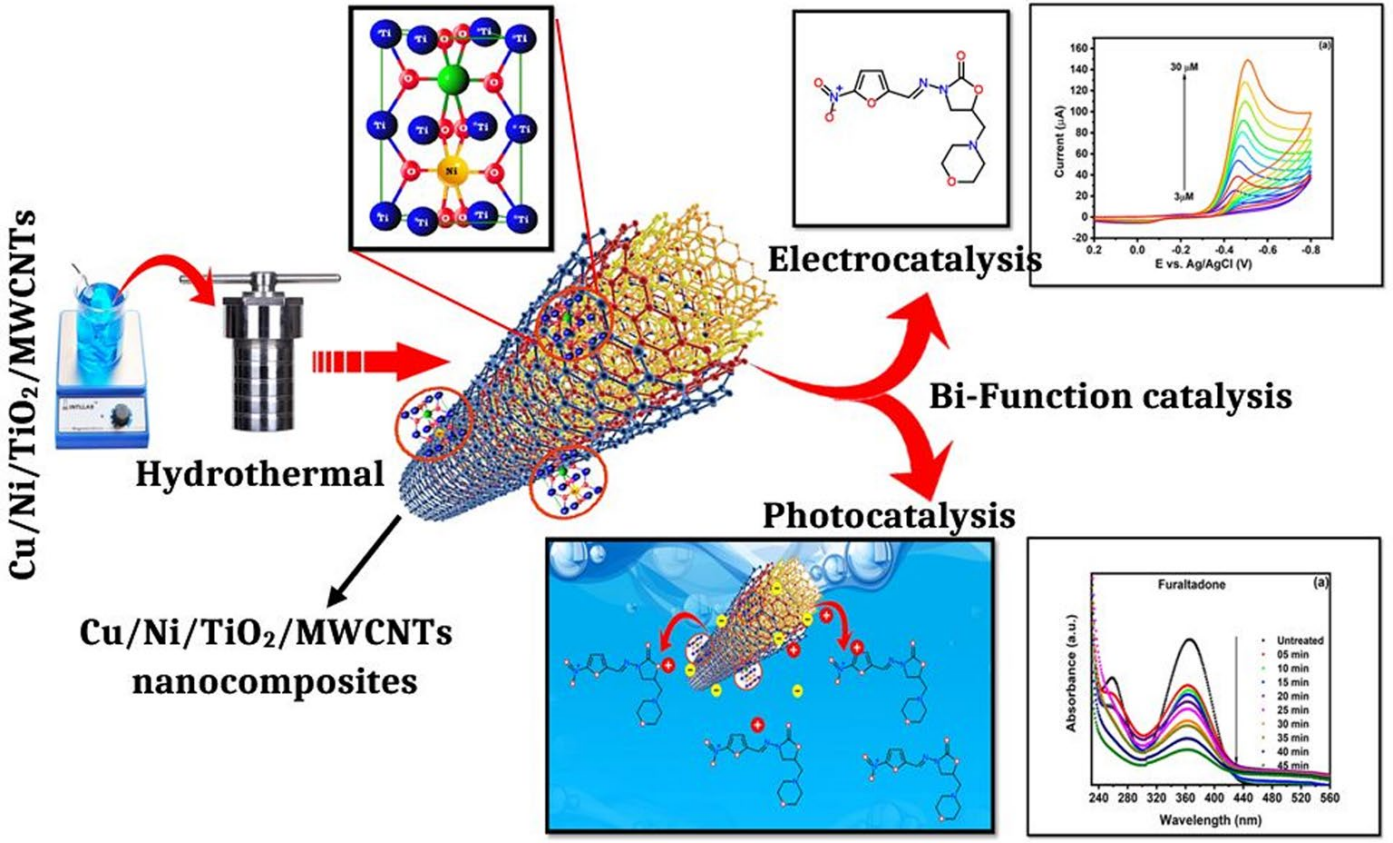
Schematic diagram for the synthesis of Cu/Ni/TiO_2_/MWCNTs nanocomposites and bi-functional applications.

In recent decades, among carbon-related materials, after the innovation of carbon nanotubes, photocatalysis-multi walled carbon nanotubes (MWCNTs) composites have attracted more attention to the scientific community due to their remarkable mechanical, electrical, and thermal properties. The synergistic effect of MWCNTs and their composite photocatalyst activity can be clarified in terms of their action as dispersing agents and adsorbents. Moreover, MWCNTs have multiple layers of superimposed graphite layers and rolled in on them to grow tubular conductive structures. The separation of the photo-generated electron–hole pairs at the photocatalysis-MWCNT interfaces might facilitate by the conductive structure^26–29^. In this work, a bi-functional novel bimetallic (Cu/Ni/TiO_2_/MWCNTs) photoanode was synthesized by simple hydrothermal methods. The synthesized nanocomposites were analyzed by various characterization techniques. As prepared nanocomposites were further studied for electrochemical sensing and photocatalytic degradation by using FLT drugs, which is shown in Fig. 1. Fortunately, we have found that the as-synthesized Cu/Ni/TiO_2_/MWCNTs nanocomposites exhibited a more active photo-electro catalyst for the electrochemical sensing of FLT, whereas its exhibit photocatalytic activity for degradation of FLT wastewater samples. The electrochemical sensing and degradation process were deeply investigated with a higher degradation rate.

## Results and discussion

### The crystalline structure and vibrational bonds of Cu/Ni/TiO_2_/MWCNTs nanocomposites

**Figure 2:**
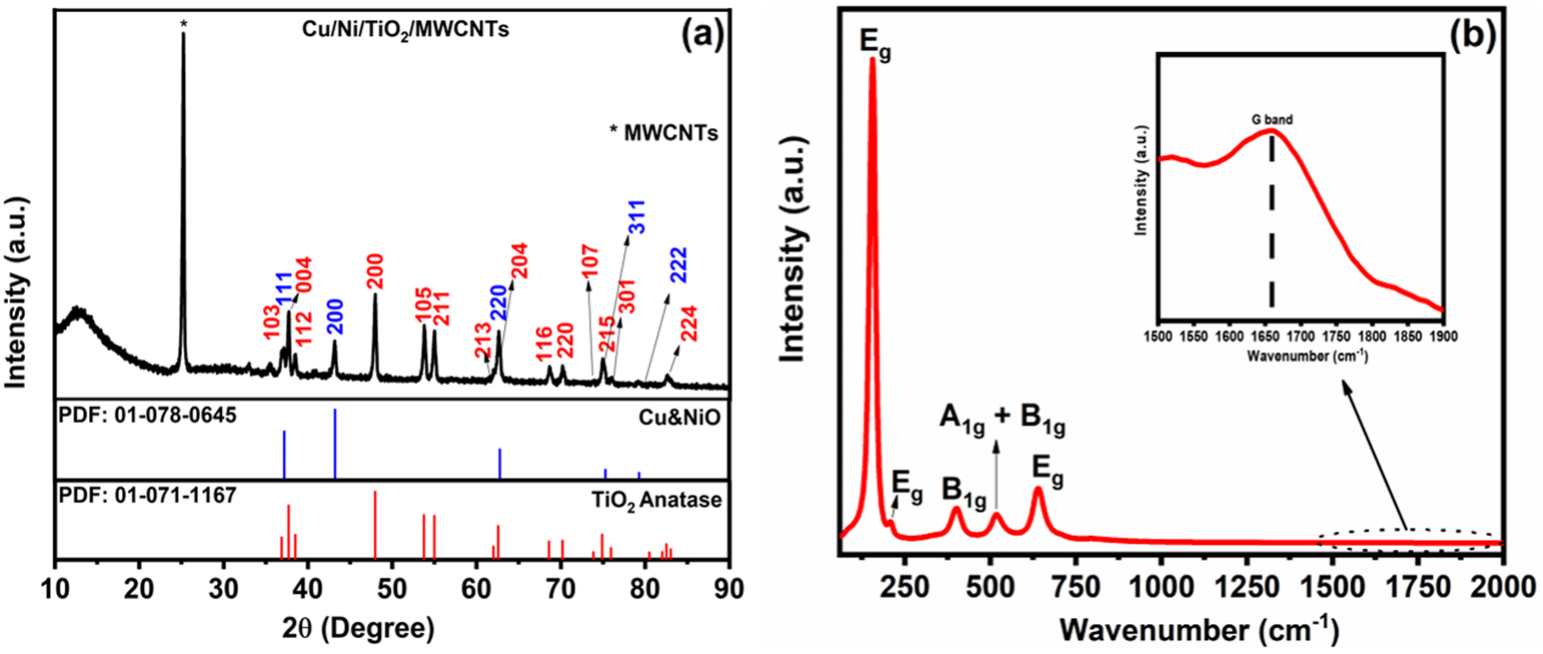
(**a**) XRD patterns, and (**b**) Raman spectra of Cu/Ni/TiO_2_/MWCNTs nanocomposites.

The hydrothermal synthesized Cu/Ni/TiO_2_/MWCNTs nanocomposites crystal structure, phase, and crystalline size were determined using XRD analysis. The synthesized nanocomposites crystal phase or XRD patterns were shown in Fig. 2a. As a result, we confirmed that the synthesized nanocomposites have a highly crystalline nature. The MWCNTs characteristics peak was also presented at 25.28 (002) (JCPDS: 41–1487). Furthermore, the higher number of TiO_2_ anatase related peaks also presented at 2θ value of 36.8, 37.6, 38.5, 47.9, 53.7, 54.9, 62.0, 62.5, 68.5, 73.8, 74.8, 75.9 and 82.5, which is corresponding to 103, 004, 112, 200, 105, 211, 213, 204, 116, 220, 107, 215, 301 and 224, (JCPDS: 01-071-1167) respectively. Simultaneously, the copper and nickel-related characteristic peaks also attributed at 2θ values of 37.21, 43.23, 62.8, 75.31, and 79.3, which is related to 111, 200, 220, 311, and 222, (JCPDS: 01-078-0645) respectively. Moreover, before MWCNT doped Cu/Ni/TiO_2_ crystalline structure was presented in Fig. S1. The anatase titanium-related peaks, copper, and nickel-related peaks were also presented. As a result, all elements successfully doped into the composites. In addition, the synthesized nanocomposite's crystalline size was calculated with help of the Scherrer equation. The calculated crystalline size of the synthesized nanocomposites (Cu/Ni/TiO_2_/MWCNTs) was approximately 25.63 nm. Therefore, the XRD analysis confirmed the formation of Cu/Ni/TiO_2_/MWCNTs nanocomposites with nanosized crystalline^28–31^.

Raman spectroscopy study is the most important technique for understanding the structural changes in the incorporation of dopant ions. The synthesized nanocomposites Raman spectra are shown in Fig. 2b. Figure 2b depicts the Raman spectra of our composites corresponding to the TiO_2_ anatase phase. Moreover, the Cu and Ni or its oxide-related secondary peaks were not observed. The high intense peak at 156 cm^−1^ can be attributed to the Eg mode of anatase TiO_2_. In addition, low intense peaks were obtained at 207 and 638 cm^−1^. The B1g and A1g + B1g modes also presented at 401 and 516 cm^−1^, respectively. Moreover, MWCNTs characteristic peaks are also attributed at 1659 cm^−1^. The symmetric stretching vibration of O-Ti–O in TiO_2_ is related to the Eg peak, the B1g modes are related with the symmetric bending vibration of O-Ti–O, and the A1g mode presents is as a result of antisymmetric bending vibration of O-Ti–O^31,32^. The G-band of MWCNTs is associated with the sp^2^-hybridized C–C bond^33^. The Cu and Ni ionic radii of Cu^2+^(0.73 Å) and Ni^2+^(0.69 Å), respectively, which is higher than the Ti^4+^ (0.64 Å), hence, the dopant ions will disturb the TiO_2_ lattice structure. The doping of Cu and Ni initiates the oxygen vacancies in the TiO_2_ lattice to maintain charge neutrality. If Cu and Ni ions doping occurs on the Ti^4+^ lattice, the pond of Ti–O-Ti will be affected and a new bond of Cu–O–Ti or Ni–O–Ti will be formed. Therefore, the Raman active modes are affected by the formation of new Cu–O and Ni–O bonds. Although Cu^2+^ and Ni^2+^ ions will disturb all Raman-active modes. A Raman study revealed that the ions were doped and MWCNTs also loaded successfully.

### SEM–EDX analysis for Cu/Ni/TiO_2_/MWCNTs nanocomposites

**Figure 3:**
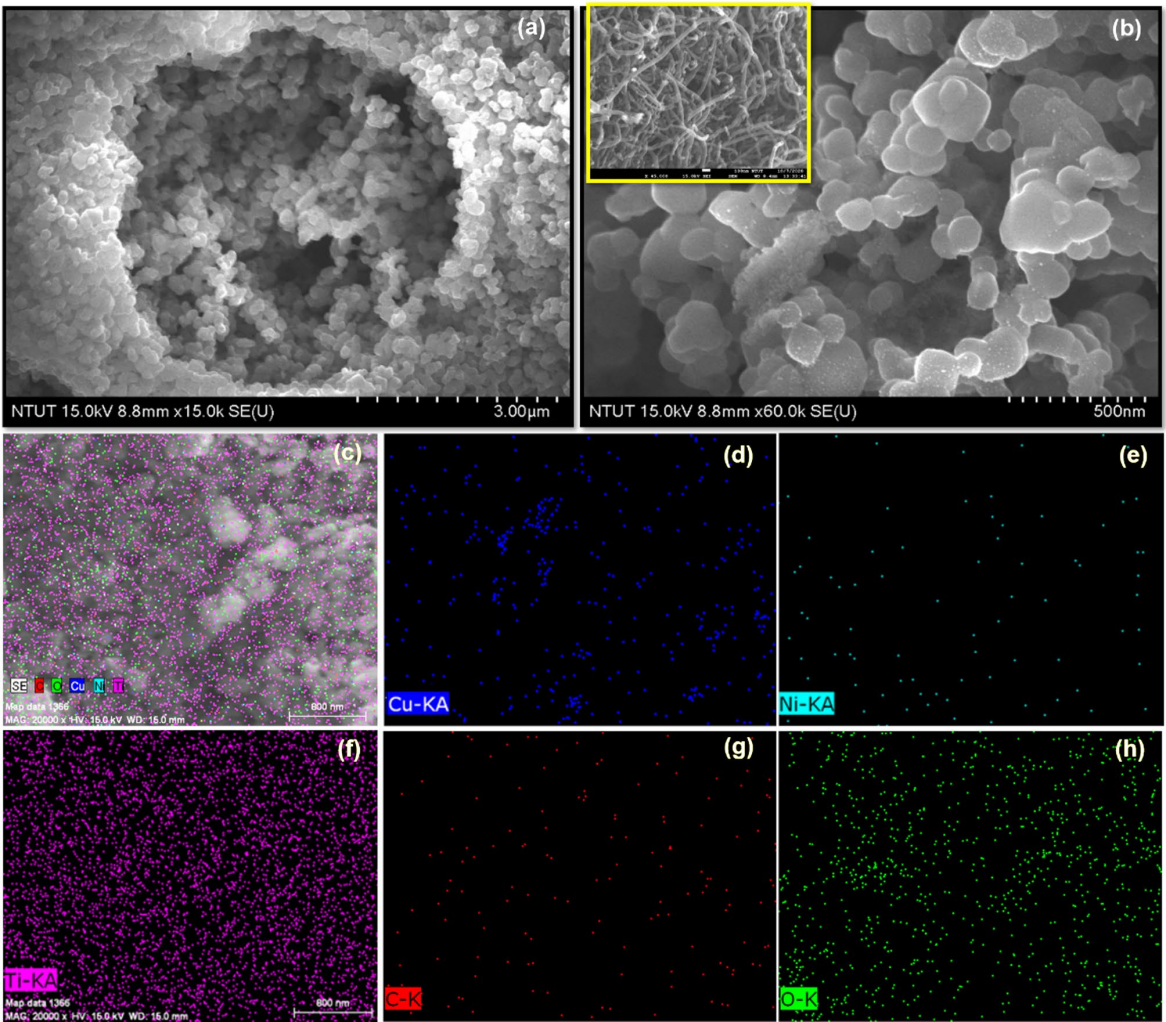
(**a**, & **b**) FESEM images of Cu/Ni/TiO_2_/MWCNTs nanocomposites, and (**c**) the combined elemental mapping of Cu/Ni/TiO_2_/MWCNTs nanocomposites (**d**) C, (**e**) O, (**f**) Cu, (**g**) Ni, (**h**) Ti.

The photo-electro catalyst properties such as transmission, the absorbance of light, and dispersion can be affected by surface morphology and particle size. Hence, the synthesized nanocomposites' surface morphology and size were observed by using SEM analysis. As an SEM analysis, the synthesized composites are grown by the spherical particles with an average particle size is ~ 60 to 80 nm. In addition, the MWCNTs images were incorporated inside the figure of Fig. 3b. Simultaneously, the Cu/Ni/TiO_2_ composites structures were also analyzed and compared to Cu/Ni/TiO_2_ /MWCNTs composites, which are screened in Fig. S2. Figures 3a,b, illustrate the Cu/Ni/TiO_2_/MWCNTs nanocomposites structure and surface morphology. EDX result along with the elemental mapping from a region of the composites confirmed the presence of all elements, which is illustrated in Fig. 3c–h. In addition, all the elements have been presented and they are uniformly distributed throughout the composites.

### XPS study

**Figure 4:**
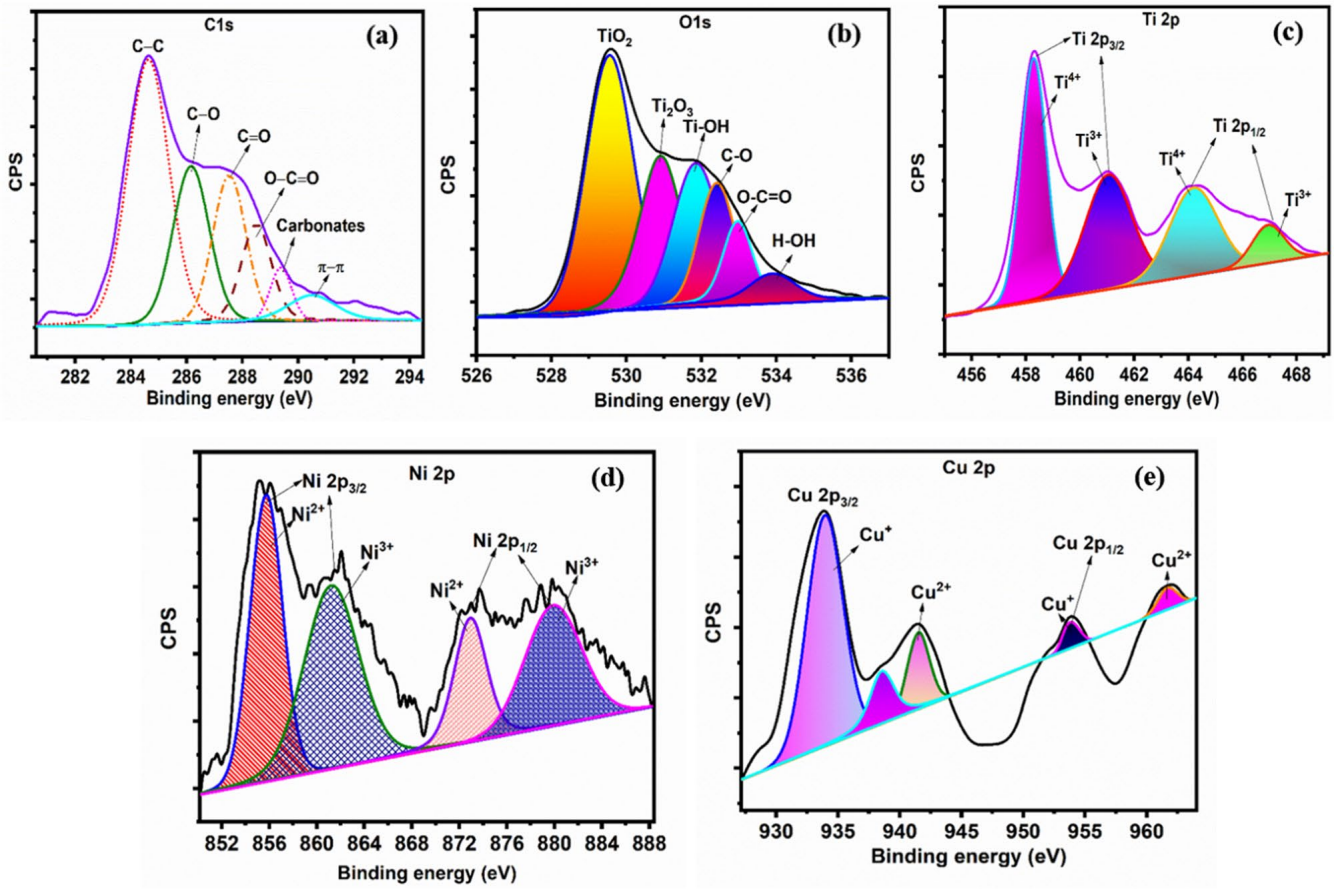
High resolution XPS spectra of Cu/Ni/TiO_2_/MWCNTs nanocomposites (**a**) C 1 s spectra, (**b**) O 1 s spectra, (**c**) Ti 2p spectra, (**d**) Ni 2p spectra, and (**e**) Cu 2p spectra.

The hydrothermal synthesized Cu/Ni/TiO_2_/MWCNTs nanocomposites surface chemistry was analyzed by using XPS analysis. The XPS is a high surface sensitive analysis and provides information about the chemical state changes of constituting species. Figure 4 shows the high-resolution XPS spectra of Cu/Ni/TiO_2_/MWCNTs nanocomposites chemicals compositions. The high-resolution spectra of XPS were corrected with a standard graphite position of 284.4 eV, and each elemental composition was fitted by using XPSPEAK41 software with a Shirley background^34^. Fig. 4a depicts the high-resolution deconvoluted spectra of C1s for Cu/Ni/TiO_2_/MWCNTs nanocomposites, which have four oxygen-related functional groups at 285.8, 287.02, 288.78, and 289.5 eV. These oxygens-related functional groups are attributed to the C–O (hydroxyl), C=O (carbonyl), O–C=O (carboxylic acid), and carbonates bonds, respectively. Moreover, the C‒C (aromatic) and π‒π interaction bonds also presented at 284.6 and 290.3 eV, respectively^34–36^. The O1s deconvoluted spectra can be shown in Fig. 4b, which have different constituents attributed to oxygen-related chemical bonds: TiO_2_, Ti_2_O_3_, Ti–OH, C–O, O–C=O, and H–OH are also presented. Therefore, the doped materials provide active sites in nanocomposites surfaces.

The Ti2p high-resolution deconvoluted spectra are shown in Fig. 4c. The doublet Ti2p_3/2_ and Ti2p_1/2_ arise from spin orbit-splitting and are attributed at 458.30, and 464.19 eV, respectively. These peaks are corresponding with Ti^4+^ in TiO_2_ lattices. In addition, the shoulder peaks were also observed at 461.04 and 466.98 eV, which corresponds to Ti^3+^ in Ti_2_O_3_ lattice. The Ti2p deconvoluted spectra indicated that both TiO_2_ and Ti_2_O_3_ are presented in the nanocomposites^37–41^. The Ni2p peaks are located at 855.6 (Ni^3+^) and 861.31(Ni^2+^) eV are due to 2p_3/2_ and 2p_1/2_, respectively (Fig. 4d), and the additional shoulder peaks also presented at around 872.8 and 879.91 eV. The presence of Ni3^+^ on the surface of nanocomposites may arise from the surface oxidation of Ni (OH)_2_^42–45^. Figure 4e depicts the Cu2p high-resolution deconvoluted spectra of the Cu/Ni/TiO_2_/MWCNTs nanocomposites have major two peaks at 933.9 and 953.8 eV due to Cu2p_3/2_ and Cu2p_1/2_, respectively. These peaks are corresponding to Cu^+^ (Cu_2_O) and Cu^2+^ (CuO) oxidation states. In addition, these peaks related shoulder peaks are observed at 953.5 and 961.6 eV. As a result, the CuO and Cu_2_O were formed in the nanocomposites^45–47^. Therefore, the formation of Ni^2+^ and Cu^2+^ are increasing the lifetime of the photo-generated electrons and lead to induce the oxygen defect resulting generation of excess electrons and various reactive species that improved the photo electrocatalytic activity.

### Electrochemical activity

#### EIS characteristics of bare SPCE and Cu/Ni/TiO_2_/MWCNTs nanocomposites fabricated SPCE

**Figure 5:**
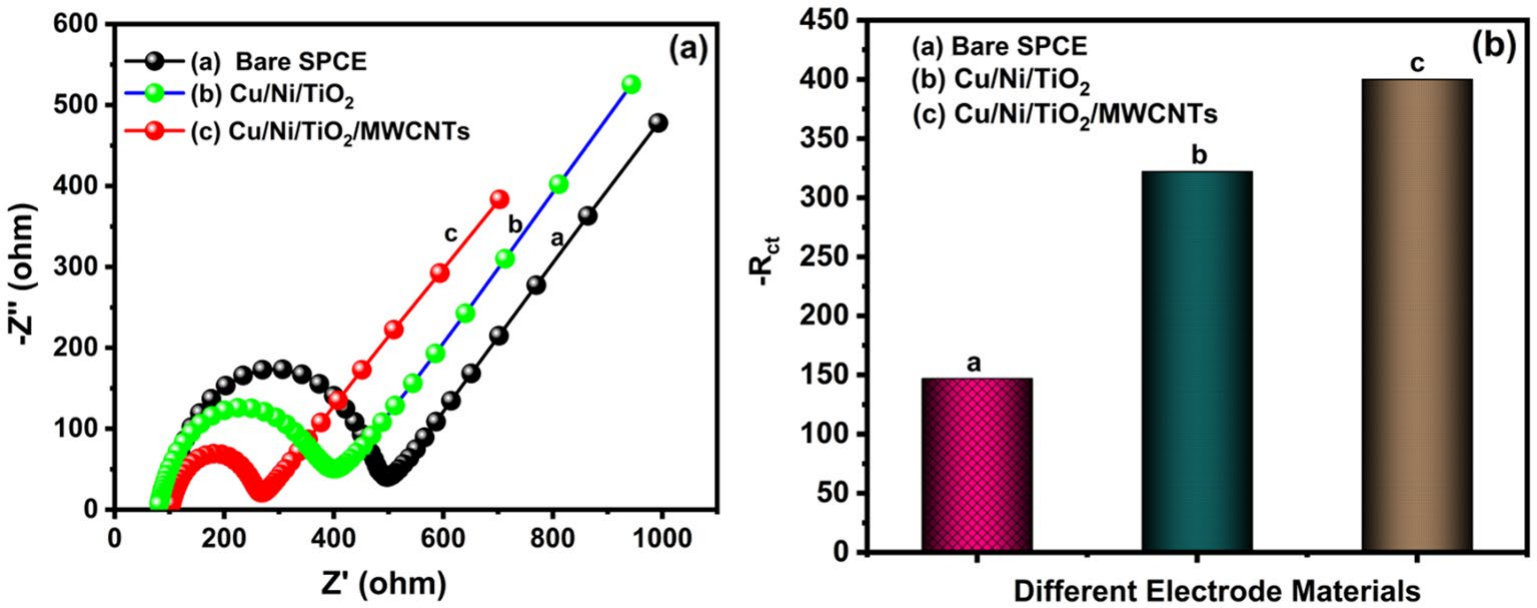
(**a**) EIS analysis of bare SPCE and Cu/Ni/TiO_2_/MWCNTs nanocomposites fabricated electrode in 0.1 M KCL at 5 mM [Fe (CN)_6_]^3−/4−^. (**b**) *R*_*ct*_ values of bare SPCE and Cu/Ni/TiO_2_/MWCNTs nanocomposites fabricated electrode.

EIS analysis is a most important electroanalytical technique, which has been widespread application in the field of research to analyzing various characteristics such as charge transfer kinetics, electrode-material interfacial resistance, mass transfer property, and diffusion coefficients, etc., Moreover, it is used to portray and monitor electrical behavior and electrochemical system sensitivity. The as-prepared electrocatalyst property of electrochemical ability is checked by using 5 mM [Fe (CN)_6_]^3^^/^^4^. Simultaneously, the frequency spectrum of 100 mHz to 100 kHz in 0.1 M KCL solution is used to obtain the Nyquist plot, and the obtained results for bare SPCE and Cu/Ni/TiO_2_/MWCNTs nanocomposites fabricated SPCE electrodes are shown in Fig. 5a. The plot has typically divided into two regions: a semicircular region and a liner zone. The semicircular zone does have a greater amplitude region, which is attributed to the charge transfer resistance (*R*_*ct*_). On the other hand, the linear zone has a lower frequency area that is linked to the diffusion region. As a result, the bare electrode and Cu/Ni/TiO_2_ composites have a higher *R*_*ct*_ value of about 322, and 398, respectively, indicating that it has poor electron transport properties. However, the Cu/Ni/TiO_2_/MWCNTs nanocomposites fabricated SPCE has lower resistance compared to bare SPCE and Cu/Ni/TiO_2_ composites the *R*_*ct*_ values also decreased to 150 Ω, (Fig. 5b) because of its catalytic properties. As a result of EIS analysis, the smaller semicircular region diameter of nanocomposites indicates low internal resistance with higher electron transfer, which confirms that the nanocomposite has higher photoelectrochemical performance compared to another one. Moreover, copper can resist both exciting holes and electrons, which is the best way to decrease the value of recombination resistance other than Ni as the only resist hole. Additionally, different n-p junctions were generated in TiO_2_ junctions due to the presence of CuO and NiO as p-type semiconductors in the TiO_2_ (n-type) structure, which can enhance the conductivity of the photoanode. As an EIS result, the Cu/Ni/TiO_2_/MWCNTs nanocomposites fabricated SPCE electrodes exhibited an excellent electrocatalytic kinetics activity compared to the bare SPCE.

#### The Cu/Ni/TiO_2_/MWCNTs nanocomposites electrocatalytic activity for the detection of FLT

**Figure 6:**
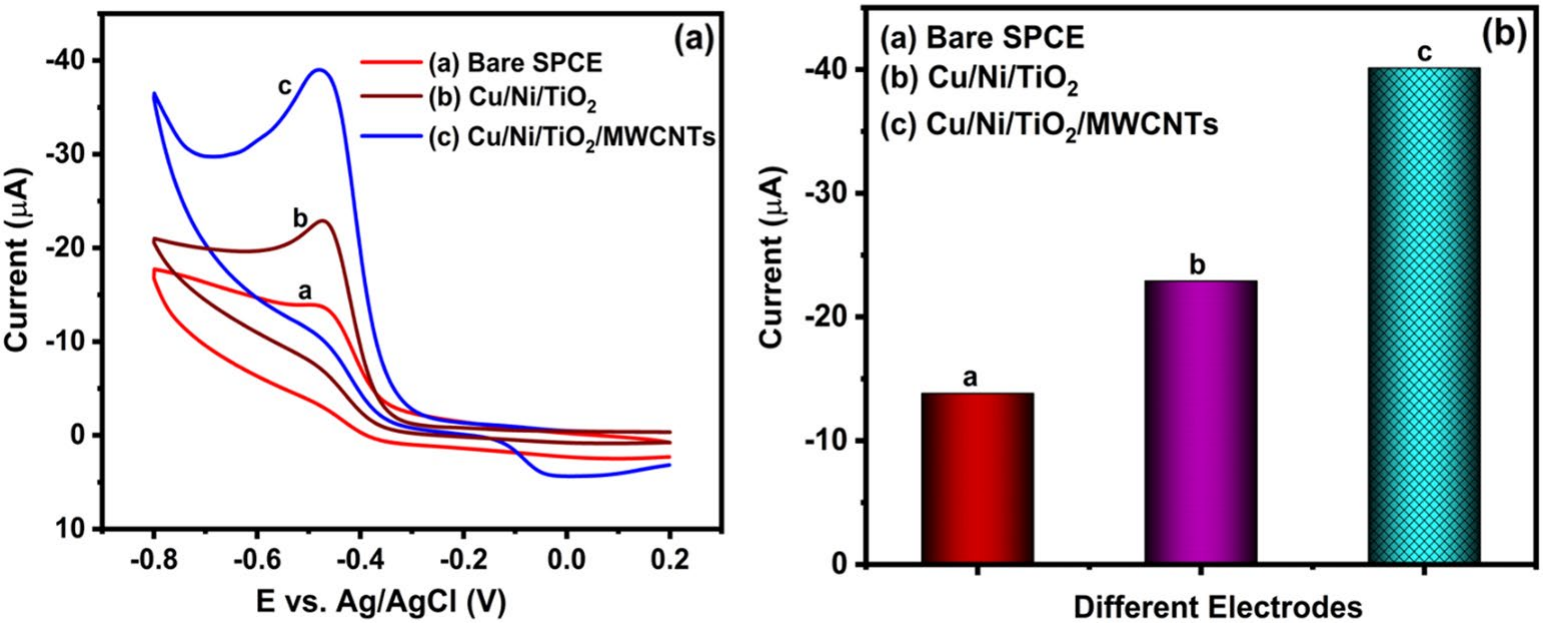
(**a**) CV plot for the electrocatalytic activity of bare SPCE and Cu/Ni/TiO_2_/MWCNTs nanocomposites fabricated electrode, and (**b**) bar diagram of bare SPCE and decorated SPCE current values.

The surface of bare SPCE and Cu/Ni/TiO_2_/MWCNTs nanocomposites modified electrodes are used to obtain the electrochemical characteristics of FLT by using CV, which is performed to presence and absence of 20 μM FT in 0.05 M PBS (pH 7.0) (N_2_-saturated) at a sweep rate of 50 mV/s (reaction window: 0.2 to − 10.8 V). Figure 6a shows the CV curves of bare SPCE, Cu/Ni/TiO_2_ nanocomposites, and Cu/Ni/TiO_2_/MWCNTs nanocomposites fabricated electrode. As a result, in bare SPCE electrode and Cu/Ni/TiO_2_ nanocomposites, a small number of reduction peaks have been observed at − 0.47 V with a lower cathodic current density of approximately − 13.91, and 22.91 μA, respectively. However, the Cu/Ni/TiO_2_/MWCNTs nanocomposites fabricated electrodes strongly pronounced reduction peaks at the same potential with a higher cathodic current density of − 39.17 μA. In addition, there is no external peaks are observed during the reverse scan in both bare SPCE and Cu/Ni/TiO_2_/MWCNTs nanocomposites fabricated electrodes. The cathodic peak's presence may be due to the direct reduction of the FLT nitro group (R-NO_2_) into the hydroxylamine group (R-NHOH). As a result, Cu/Ni/TiO_2_/MWCNTs nanocomposites have greatly reduced the FLT into a hydroxylamine group, hence, the higher cathodic current signal is observed compared to unmodified SPCE, which is shown in Fig. 6b. The modified Cu/Ni/TiO_2_/MWCNTs nanocomposites surface is highly active and has excellent electrocatalytic activity against the FLT nitro group for strong electrochemical interaction. Hence, the Cu/Ni/TiO_2_/MWCNTs nanocomposites have been chosen as a working electrode material for the electrochemical measurement of FLT because of its electrical conductivity, electron mobility, and suitability for a green environment. The Cu/Ni/TiO_2_/MWCNTs nanocomposites have more vacancies and the highest occupied molecular orbital (HOMO) electron of the composites further transferred into the lowest unoccupied molecular orbital (LUMO) level of FLT resulting reduction process has occurred.

#### Influence of electrolyte pH

**Figure 7:**
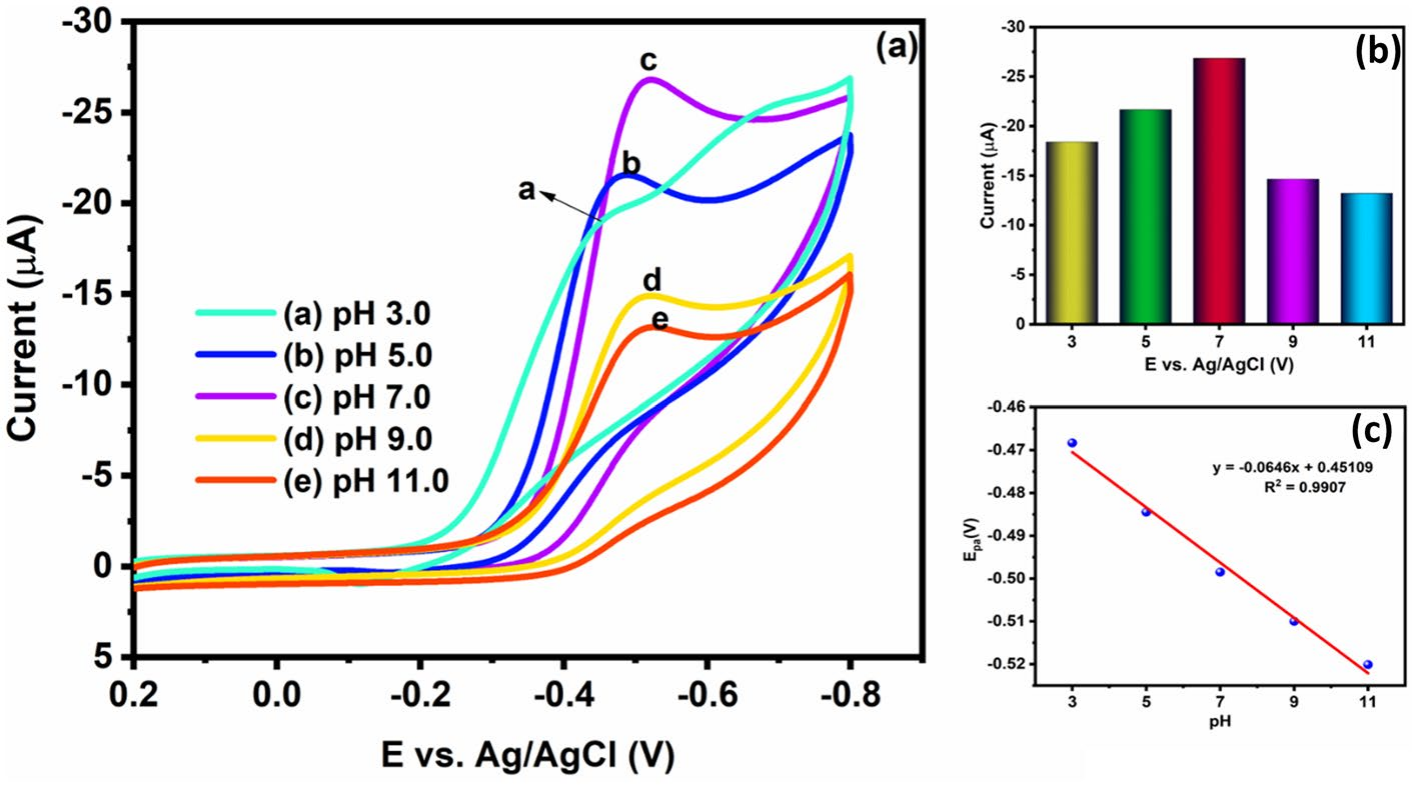
(**a**) CV results of Cu/Ni/TiO_2_/MWCNTs nanocomposites with various pH values. (**b**) peak current for various pH values, and (c) linear coefficient pH vs potential.

The electrochemical behavior of FLT is affected by the electrolyte solution pH. Hence, the effect of pH was investigated on the reduction of FLT with the Cu/Ni/TiO_2_/MWCNTs nanocomposites to fixed (20 μM) concentration of FLT with different pH such as 3, 5, 7, 9 and, 11 by using CV (Fig. 7a). The FLT reduction cathodic peak current was increased by increasing the electrolyte pH from 3.0 to 7.0, whereas the cathodic peak current is decreased further increasing the solution pH above 7.0 (such as 9.0 and 11.0) (Fig. 7a and b). Concurrently, the linear plot shows the linear relationship with R^2^ = 0.9907, which is shown in Fig. 7c. When the pH was 7.0, FLT^±^ content increased rapidly, with a subsequent decrease in FLT^−^ and the cathodic peak current attains a maximum value under the reaction of both hydrogen bonding and electrostatic attraction. However, the pH is increased above 7.0, the FLT^−^ concentration increased gradually hence, the excess FLT^−^ content induced electrostatic repulsion with Cu/Ni/TiO_2_/MWCNTs nanocomposites electrode surface. Additionally, the Cu/Ni/TiO_2_/ MWCNTs nanocomposites and FLT^−^ hydrogen bonding is hypothesized. Therefore, the hydrogen bonding and electrostatic repulsion action are combined under alkaline conditions resulting in a rapid decrease of FLT cathodic peak current compared to acid ones^48^. Moreover, the FLT reduction cathodic maximum current is observed at pH 7.0 due to the contribution of excited electrons and the electron mobility of the Cu/Ni/TiO_2_/MWCNTs nanocomposites at its high speed. As a result, pH 7.0 is the most suitable electrolyte solution pH for studied about the electrochemical activity of FLT reduction. Hence, pH 7.0 is the most preferred optimum electrolyte pH for further electrochemical studies of FLT.

#### Effect of FLT concentration

**Figure 8:**
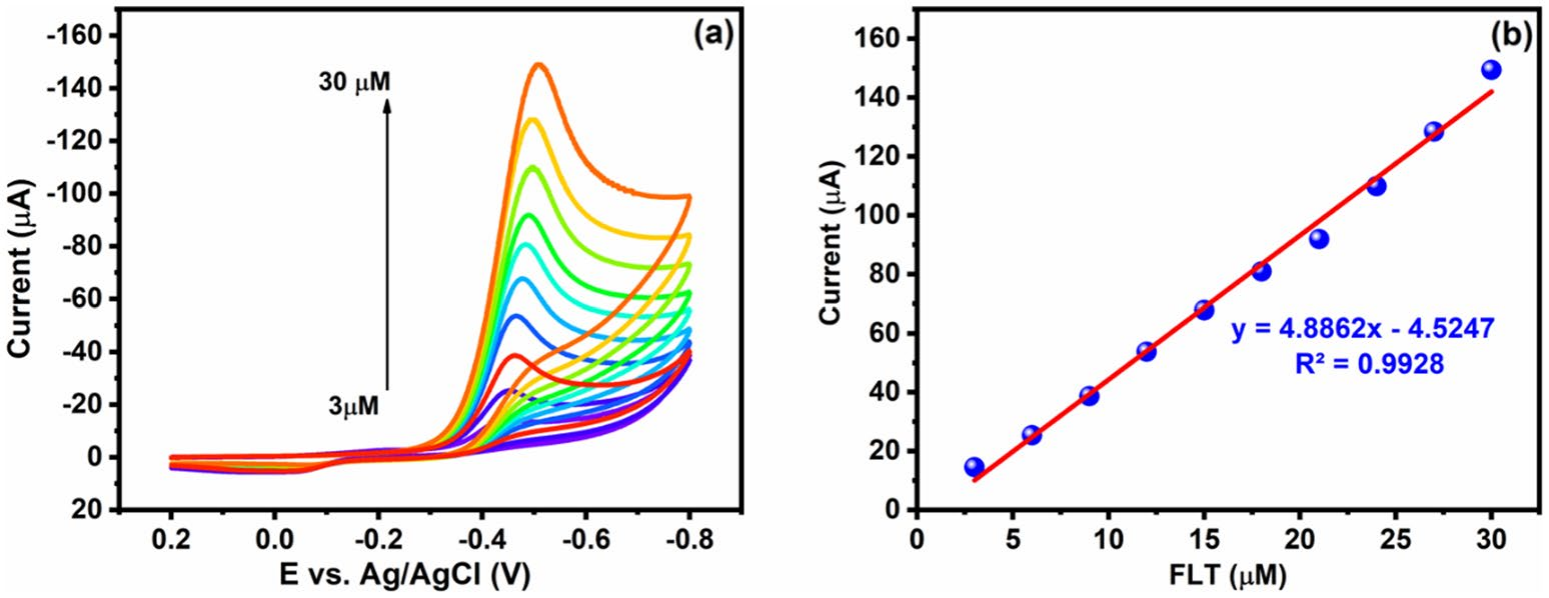
(**a**) Cu/Ni/TiO_2_/MWCNTs nanocomposites with different concentrations of FLT reduction CV curves presence of 0.05 M PBS (**b**) linear regression of peak currents *vs* different concentrations of FLT.

The antifouling property of desired materials is a more important thing for electrochemical studies, which are carried out with various concentrations of FLT. Figure 8a depicts the Cu/Ni/TiO_2_/MWCNTs nanocomposites antifouling property to different mole concentrations (10–150 μM) of FLT with a constant scan rate (0.05 V/s) in 0.05 M of PBS (pH 7.0). In addition, the CV analysis revealed the Cu/Ni/TiO_2_/MWCNTs nanocomposites antifouling effect with the various concentration of FLT. Moreover, the linear plot is obtained when the cathodic peak current was plotted against the different concentrations of FLT. Concurrently, the linear plot shows the linear relationship with R^2^ = 0.9928, Ipc (μA) = − 4.8862 μM–4.5247, which is shown in Fig. 8b. Undoubtedly, the cathodic reduction peak of FLT was obtained, and reduction of FLT nitro group into hydroxylamine group has observed.

#### Effect of Scan rate

**Figure 9:**
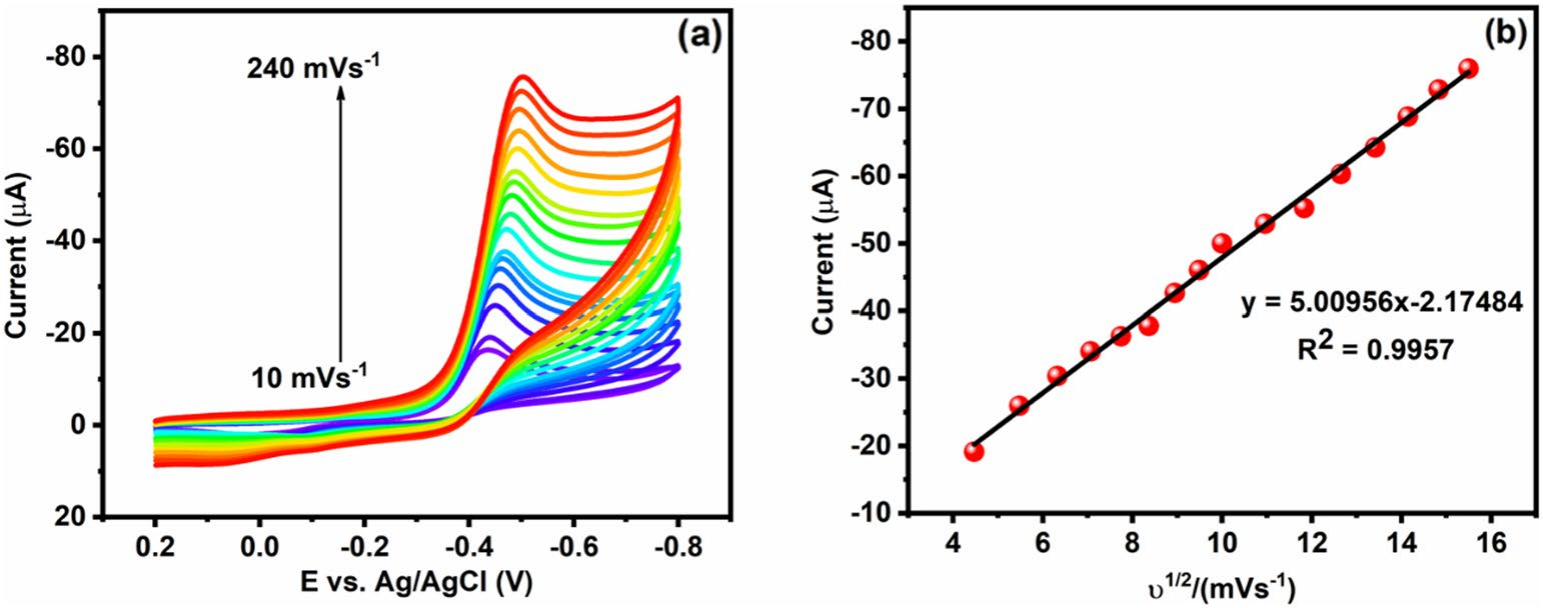
(**a**) Cu/Ni/TiO_2_/MWCNTs nanocomposites with different scan rates of CV curves presence of 0.05 M PBS with 20 μM of FLT. (**b**) linear regression of peak currents *vs* square root of the scan rates.

In electrochemical techniques scan rate is an important parameter for studying the kinetic studies of FLT from the cathodic peak current and scan rate on the electrode surface. The influence or effect of scan rate is evaluated by Cu/Ni/TiO_2_/MWCNTs nanocomposites with different scan rates 10—240 mV s^−1^ and 0.05 M PBS solution containing 20 μM of FLT. As shown in Fig. 9a, the cathodic peak currents linearly increased with increasing the scan rates and peak currents mainly depend upon the scan rates. Furthermore, the cathodic peak current is directly proportional to each scan rate. The linear plot is obtained when plotting the square root of scan rate *vs* peak current, and the linear value is I_pc_ (μA) = − 5.00956*v*^2^ + 2.1748 and R^2^ = 0.9957. The obtained results portray the surface-controlled process and it induces the FLT nitro group reduction by the Cu/Ni/TiO_2_/MWCNTs nanocomposites modified electrodes. As a result, the prepared nanocomposite electrodes have high electrochemical characteristics due to their high electron mobility, porosity, and excellent electrocatalytic ability. Moreover, the electron transfer is adequate to reduce the FLT nitro group on Cu/Ni/TiO_2_/MWCNTs nanocomposites, which is revealed that in Fig. 9b.

#### Determination of FLT using DPV analysis

**Figure 10:**
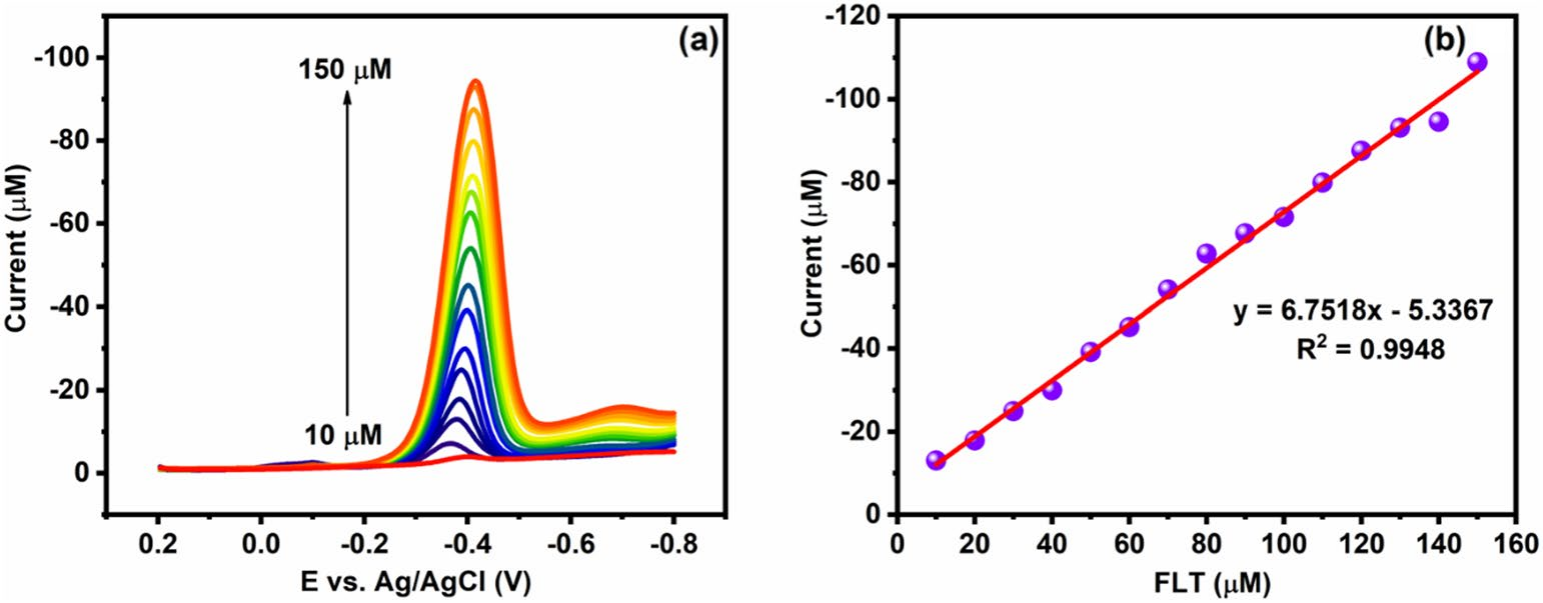
(**a**) DPV techniques for the detection of FLT with various concentrations from 10 to 150 μM using Cu/Ni/TiO_2_/MWCNTs composites, and (**b**) linear calibration plot for reduction peak current versus FLT concentration.

DPV techniques are one of the most commonly used methods for detecting analytes in flow systems, as well as the in vivo analysis of blood and DNA. In addition, DPV is more favorable for the quick analysis of a single analyte. Indeed, DPV has an enormous benefit over the other analytical technique that can diminish the effect of capacitive current and enhance the signal-to-noise ratio by attenuating the background current^49^. Therefore, DPV techniques are used to observe the modified electrode's sensitivity, detection limit, and linearity. In this study, the modified electrodes used obtained the concentration of FLT as shown in Fig. 10a. Figure 10a depicts the DPV analysis of FLT reduction on Cu/Ni/TiO_2_/MWCNTs nanocomposites in 0.05 M of PBS with different concentrations of FLT (10–150 μM). Concurrently, the concentration of R-NO_2_ increased as well as the reduction current also increased linearly and, it depicts an excellent coefficient value R^2^ = 0.9948 (Fig. 10b). The linear plot has been used to observe the modified electrode's electrochemical parameters such as LOD and sensitivity. The modified electrode's LOD limit can be calculated using the following expression^49^.

$$LOD = \frac{{3 \times standared\;deviation\left( {SD} \right)}}{slope}{{\mu {\text{A}}\mu {\text{M}}}}^{ - 1} {\text{cm}}^{ - 2}$$

Hence, the calculated LOD and sensitivity values of the modified electrode are 0.0949 μM and 1.9288 μA μM^−1^ cm^−2^. As a result, the modified electrodes have an excellent electrochemical ability towards FLT reduction.

#### Stability, repeatability, and reproducibility

**Figure 11:**
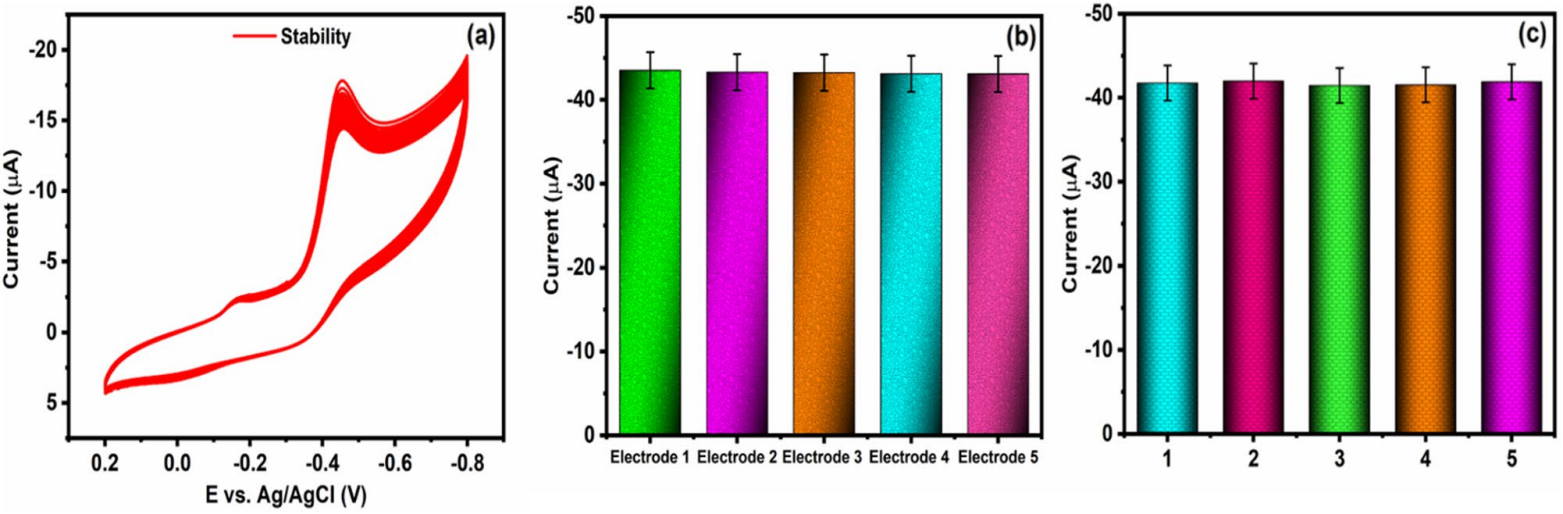
Stability, reproducibility, and repeatability studies of Cu/Ni/TiO_2_/MWCNTs nanocomposites modified electrode. (**a**) stability studies, (**b**) bar diagram of reproducibility for different electrodes, and (**c**) bar diagram of repeatability for the same electrode.

The nanocomposite modified electrodes were scrutinized for stability, reproducibility, and repeatability studies by using CV techniques with help of 20 μM in 0.05 M of PBS (pH 7.0) (Fig. 11a,b,c). Moreover, five independent nanocomposites modified SPCE is used to study the reproducibility studies. Concurrently, for repeatability experiments, five individual experiments with the same electrode were performed. As a result, the obtained peak current at each study is significantly similar, and thus nanocomposite modified electrodes prove better repeatability and reproducibility ability. Furthermore, the nanocomposites modified electrode's cyclic stability was also observed by the CV technique with FLT (20 μM) along with PBS (pH 7.0) and a scan rate of 50 mV s^−1^ (Fig. 11a). The obtained results revealed that the current loss from the 1st cycle to the 100th cycle was estimated to be less than 5%, which embraces stability for electrochemical performances of FLT towards nanocomposites modified electrode.

#### FLT Real samples analysis

The Cu/Ni/TiO_2_/MWCNTs nanocomposites modified electrodes ability is evaluated with help of the FLT real sample analysis process. Furthermore, the real sample analysis is performed by DPV technique with help of pond water and tap water, which is picked from the Taipei riverside and our laboratory tap water. Before the experiment, the obtained water samples are centrifuged (at 2000 rpm) and filtered with Whatman filter paper to eliminate the solids and other wastes. Before the analysis, a known amount of FLT is added into the FLT free water samples. Concurrently, the analysis of the real samples indicates that the FLT concentration which is lower than the detection limit. The real samples analysis is given in Table 1. As a result, the modified electrochemical sensor has proved its electrochemical potential application to the analysis of the real sample.

**Table 1:** Different real water samples analysis using Cu/Ni/TiO_2_/MWCNTs nanocomposites.

Samples	Added (μM)	Found (μM)	Recovery (%)	RSD (%)
Tap Water	10	9.32	93	2.19
	20	19.68	98.4	2.47
	30	29.24	97.4	4.1
Pond water	10	9.14	91	4.57
	20	18.63	93.1	2.51
	30	29.02	96.7	2.09

### Photocatalytic studies

#### Furaltadone degradation capacity under Visible light

**Figure 12:**
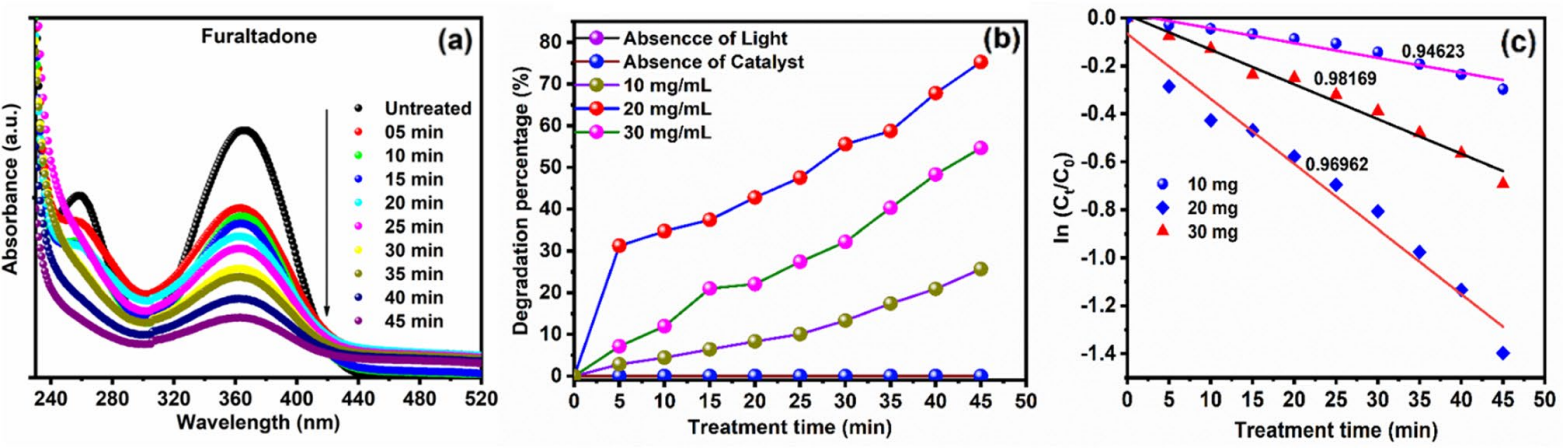
The synthesized Cu/Ni/TiO_2_/MWCNTs nanocomposites photocatalytic activities using FLT (**a**) Absorbance spectrum of FLT before and after the treatment process, (**b**) Degradation percentage of FLT with various concentration function of treatment time (**c**) FLT degradation first-order kinetic equation.

The photocatalytic degradation of furaltadone is studied with and without Cu/Ni/TiO_2_ NPs loaded MWCNTs nanocomposites under visible light irradiation as a function of various treatment conditions such as catalyst concentration and treatment time. The degradation/decolorization of furaltadone is measured using their maximum absorbance intensity of 360 nm (Fig. 12a). After the treatment, the maximum absorbance intensity of furaltadone is decreased. However, there is no degradation recorded during the treatment for the absence of catalyst and absence of light source, which means it is the catalyst and light-free treatment. Moreover, in optimum concentrated Cu-Ni-TiO_2_ (20 mg) nanocomposites have a lower degradation percentage compared to Cu-Ni-TiO_2_ loaded MWCNTs nanocomposites, which is presented in Fig. S3a,b. In addition, the furaltadone aqueous solution's maximum absorbance intensity is simultaneously decreased in the treatment of Cu-Ni-TiO_2_ loaded MWCNT under visible light irradiation. Therefore, the higher degradation efficiencies of furaltadone aqueous solution were obtained at 75% in 50 min of treatment time (Fig. 12b). It has been revealed that the Cu-Ni-TiO_2_ loaded MWCNT NPs may significantly enhance the visible light degradation performance. However, the photocatalytic assisted degradation of furaltadone efficiency is decreased when photocatalysis concentration was decreased low (10 mg) and higher (30 mg) concentration of photocatalysis, respectively. Furthermore, the higher degradation efficiencies of furaltadone were achieved in the photocatalysis concentration of 20 mg. The furaltadone degradation efficiencies is obtained for 20 mg > 30 mg > 10 mg. In addition, degradation of furaltadone is found to be followed by a first-order kinetic reaction (Fig. 12c). The influence of catalysis concentration on the visible light assisted photocatalytic degradation rate constant is described by pseudo-first-order kinetics model^50^.

$$\ln (C_{t} /C_{0} ) = - kt$$

where *k* is the kinetic rate constant whereas *C*_*t*_ and *C*_0_ are denoted as before and after the treatment process of furaltadone concentration, respectively. The photocatalytic treatment times are denoted as t. Therefore, the observed results show that the degradation rate of furaltadone using 20 mg Cu/Ni/TiO_2_/MWCNTs nanocomposites as photocatalyst is higher than the other concentration of photocatalysis.

#### Photocatalytic mechanisms

**Figure 13:**
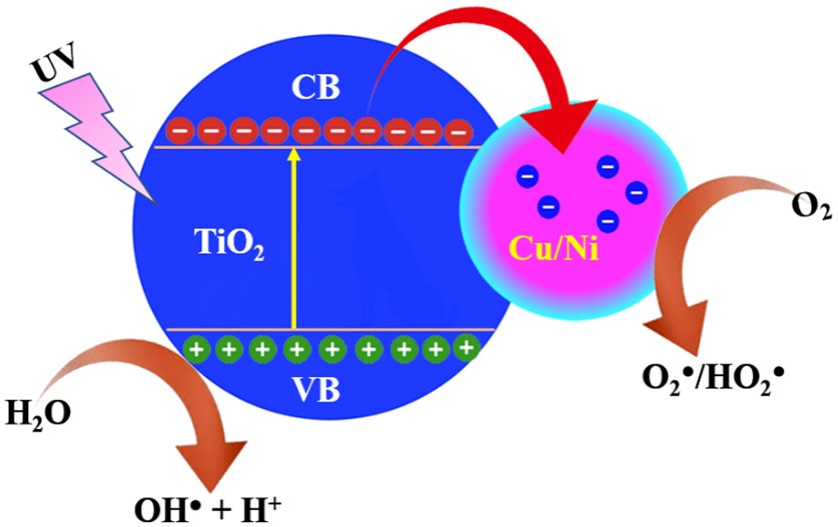
A possible electron transfer reaction mechanism during the photocatalytic degradation process.

Based on the literature survey the undoped or pure TiO_2_ photocatalysis has a wide bandgap, whereas it is active under UV lights only. Therefore, the dopant ions were introduced to the TiO_2_ crystal lattice for enhanced the physicochemical properties. On the other hand, in the case of Cu and Ni-doped TiO_2_ photocatalysis, the introduction of Cu^2+^ and Ni^2+^ with a valance bandgap less than that of Ti^4+^ (Ni^+^ + O_2(ads)_ → Ni^2+^ + e^-^, Cu^+^ + O_2(ads)_ → Cu^2+^ + e^−^, Ti^4+^ + e^−^ → Ti^3+^)^38–43^ lattice will generate an oxygen defect acting as an energetic location for organic molecules dissociation on the TiO_2_^−^ surface. In the degradation process to proceed, the generating electron–hole lift-times must be higher or long enough. Hence, this process gives more chance for the generation of charge carriers to attain the catalyst surface to interact with the adsorbed organic effluents. In the case of Cu and Ni-doped samples, the generated photo-induced electrons will be ejected by the induced surface defects in the TiO_2_ lattice. Hence, this process leads to an enhance in the electron–hole pair lifetime, which improved the possibility of reactions of reactive species generations (Yang et al., 2010). Yu et al. reported that Ni ions doped TiO_2_ catalysts have higher photocatalytic activity due to Ni^2+^ ions being successfully subjected to Ti^4+^ lattice because Ni^2+^ ionic radius is similar to that of Ti^4+^. When visible ray’s incident on TiO_2_ surface with the energy of photons is higher or equal than its bandgap, energetic electrons transfer valance band (VB) to conduction band (CB) (Fig. 13).

The excited electrons could be interacting with Ni^2+^ to reduce Ni^+^. Subsequently, Ni^+^ could be oxidized to Ni^2+^, when electrons absorbed O_2_ on the surface of TiO_2_. In addition, Ni^+^ ions interact with neighboring surface Ti^4+^ and then lead to exchange electron transfer, that transfer electron strongly interacts with organic molecules to degrade it. Moreover, Ni acts as a photogenerated electron trapper in TiO_2_ lattice. Therefore, the electron and holes generated more reactive species such as OH, O_2_^−^ and H_2_O_2_, which are the major components for the degradation of organic compounds. These species formation mechanisms are given below^51^.

$${\text{TiO}}_{{2}} + {\text{h}}\nu \to {\text{e}}_{{{\text{cb}}}}^{ - } + {\text{h}}_{{{\text{vb}}}}^{ + }$$

$${\text{Ni}}^{{{2} + }} + {\text{e}}_{{{\text{cb}}}}^{ - } \to {\text{Ni}}^{ + }$$

$${\text{Ni}}^{ + } + {\text{O}}_{{{2}({\text{ads}})}} \to {\text{Ni}}^{{{2} + }} + {\text{O}}_{{2}}^{ - }$$

$${\text{Ni}}^{ + } + {\text{Ti}}^{{{4} + }} \to {\text{Ni}}^{{{2} + }} + {\text{Ti}}^{{{3} + }}$$

$${\text{Ti}}^{{{3} + }} + {\text{O}}_{{{2}({\text{ads}})}} \to {\text{Ti}}^{{{4} + }} + {\text{O}}_{{2}}^{ - }$$

$${\text{Cu}}^{{{2} + }} + {\text{e}}^{ - } \to {\text{Cu}}^{ + }$$

$${\text{Cu}}^{ + } + {\text{O}}_{{2}} \to {\text{Cu}}^{{{2} + }} + {\text{e}}^{ - }$$

$${\text{e}}^{ - } + {\text{O}}_{{2}} \to {\text{O}}_{{2}}^{ - }$$

$${\text{h}}^{ + } + {\text{H}}_{{2}} {\text{O}} \to {\text{OH}}^{ \cdot } + {\text{H}}^{ + }$$

$${\text{Cu}}^{ + } + {\text{H}}_{{2}} {\text{O}}_{{2}} \to {\text{Cu}}^{{{2} + }} + {\text{OH}}^{ - } + {\text{OH}}^{ \cdot }$$

$${\text{Cu}}^{{{2} + }} + {\text{H}}_{{2}} {\text{O}} \to {\text{Cu}}^{ + } + {\text{OH}}^{ \cdot } + {\text{H}}$$

$${\text{2O}}_{{2}}^{ - } + {\text{2H}}^{ + } \to {\text{H}}_{{2}} {\text{O}}_{{2}} + {\text{O}}_{{2}}$$

$${\text{H}}_{{2}} {\text{O}}_{{2}} + {\text{e}}^{ - } + {\text{H}}^{ + } \to {\text{OH}}^{ \cdot } + {\text{H}}_{{2}} {\text{O}}$$

Furthermore, copper (Cu^2+^) also acts as an electron scavenger, which induces electron–hole pair separation. The ionic radius of the Cu^2+^ (0.73 Å) is nearly the same as the Ni^2+^(0.72 Å) and Cu^2+^ could be easily doped into the TiO_2_ lattice due to their ionic radii. Moreover, Cu plays as an acceptor of the impurities in Ti^4+^ lattice and restricts the recombination of photogenerated electron–hole pairs. Visible light rays’ incident on the surface of the TiO_2_ the photogenerated electron–hole pairs are generated, when incident photon energy was higher or same of the TiO_2_ CB. Indeed, the bandgap of TiO_2_ is decreased with doping of transition metal ions (Cu^+^ and Ni^+^). Simultaneously, the photogenerated electron–hole pairs interact with copper to generate copper ions and superoxide onions, and the formation mechanism is given in the above equation. Therefore, the Cu^2+^ and Ni^2+^ ions can enhance the photocatalytic ability of TiO_2_ nanoparticles loaded MWCNT for degradation of furaltadone organic pollutants. Concurrently, MWCNTs play a major role in the degradation process, because its acts as efficient adsorption of target pollutant to improve the photocatalytic degradation efficiency rates. When the photocatalysis is modified by the MWCNTs, the catalytic ability of Cu/Ni/TiO_2_ is enhanced. The contribution of nanotubes in Cu/Ni/TiO_2_/MWCNT composites is increasing the recombination time for electron–hole pair, which means suppressed the photogenerated electron–hole pair recombination. Moreover, the delocalized π-structure characteristic of MWCNTs has aided this, which enhances the transfer of electrons and can be an outstanding electron acceptor causing electron–hole trapping. Thus, improved by the excellent electrical and optical properties of Cu/Ni/TiO_2_/MWCNTs surface. Simultaneously, photocatalysis surface adsorbed O_2_ was reduced to O_2_^−^ by e^−^, which then converted into more active hydroxyl radicals. Hence, the active reactive species effectively attacked persistently to the target organic pollutants and are enhanced the degradation efficiencies^42–47^. However, the lower and higher concentration of photocatalysis degradation efficiencies was decreased due to maybe the presence of less or more reactive species. In addition, the higher concentration of photocatalysis (30 mg), which could be attributed to the shadowing effect. Moreover, the high turbidity is occurred due to the concentration of photocatalysis, which restricts the light radiation penetration depth. The photocatalysis concentration increased the Cu^2+^ and Ni^2+^ concentration also to high, which could serve as the recombination centers that restrict the generation of photogenerated holes. Hence, the ions enhance the redox process because the photogenerated hole trapping sites is decreased^45^.

#### Effect pH

**Figure 14:**
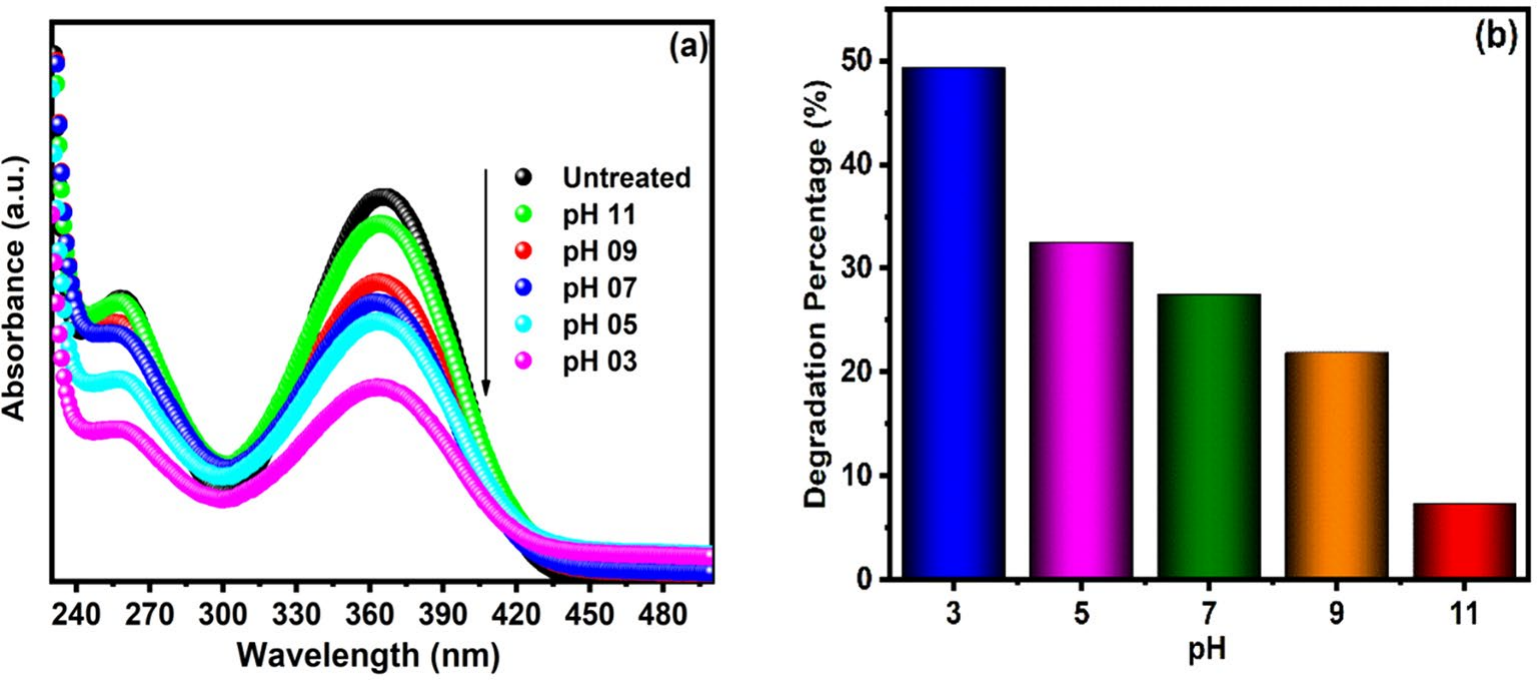
The effect of pH for degradation of FLT by Cu/Ni/TiO_2_/MWCNTs nanocomposites, (**a**) Absorption spectrum of FLT with different pH values, and (**b**) degradation percentage of FLT with different pH.

The assessment of pH effects on the efficacy of the photocatalytic degradation process is a very complicated task. Three possible reaction mechanisms can involve the dye degradation, (1) conducting band electron directly contribute the reduction process, (2) the positive hole initiating the oxidation process, and (3) hydroxyl radical attack. Those each reaction can mainly depend on the pH and substrate nature. If the solution pH is modifying the electrical double layer of the solid electrolyte interface consequently affects the catalyst's adsorption–desorption processes and the separation of electron–hole pairs. Moreover, TiO_2_ depicts an amphoteric characteristic so that positive or negative charge carriers can be developed on its surface. Hence, the pH variation can influence the adsorption of organic molecules onto the TiO_2_ surfaces^40–42^. To study the effect of pH, the photocatalytic assisted degradation of FLT by Cu/Ni/TiO_2_/MWCNTs nanocomposites was performed at various pH values. The FLT degradation is higher in acidic conditions compared to alkaline conditions (Fig. 14a,b). As a result, the pH changes are affecting the photocatalysis surface properties such as adsorption–desorption behavior and surface-charge properties. In acidic conditions, the TiO_2_ surface is positive charges is presented for absorption of negatively charged FLT initiates the degradation reaction, and higher degradation efficiency is observed. In contrast, in alkaline nature, the absorption of FLT on the nanocomposite surface decreases due to the repulsion between the FLT organic molecules into negatively charged photocatalyst surface and resulting in lower photocatalytic degradation efficiencies.

#### GC–MS analysis of Furaltadone

**Figure 15:**
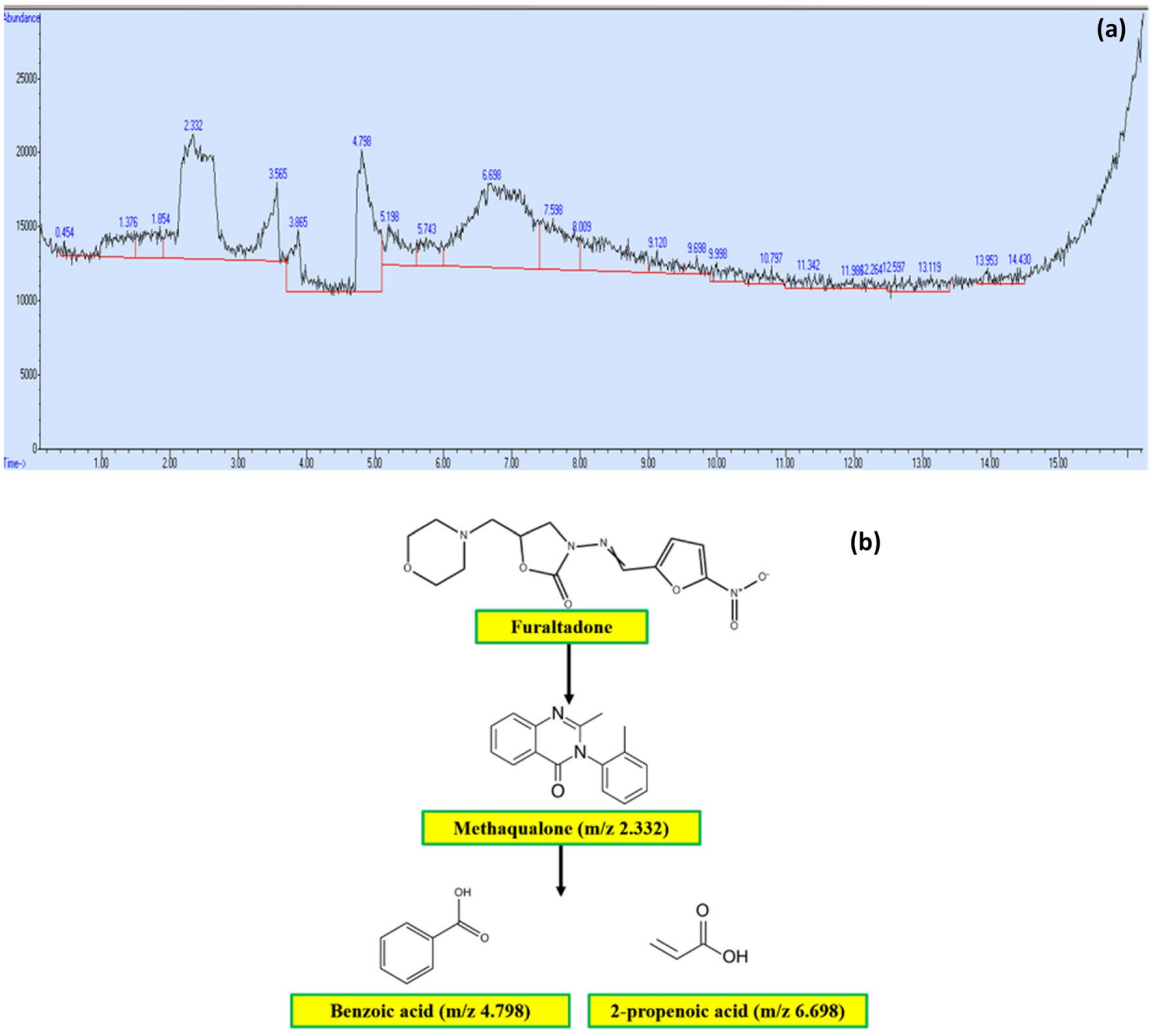
(**a**) GC chromatogram of FLT showing degradation and degradation products, and (**b**) proposed degradation pathway depicts the intermediates after photocatalytic degradation of FLT.

The GC–MS technique confirmed the degradation of FLT as showed by MS spectra of mineralized by-products in the GC chromatogram. After the decomposition (m/z values), the mineralization pathway of FLT is proposed based on the Fragmentation pattern. The FLT samples were collected after the degradation process and diluted with ethanol in the concentration of 500 ppm for GC–MS analysis. The GC–MS analysis and intermediates were depicted in Fig. 15a,b. The Figure revealed that the degradation of FLT drugs under visible light irradiation. The degradation of FLT proceeds through the breaking of their molecules by reactive radicals which are generated as a result of various photoreactions while splitting up into various intermediates. These intermediates are smaller organic molecules with low molecular weight. Therefore, it could be expressed that the first step for mineralization is adsorption of FLT molecules on nanocomposites, followed by reductive degradation/decomposition of parent molecules bond to non-toxic organic molecules, thus leading to degradation of organic compounds.

## Conclusions

In conclusion, we have studied novel nanocomposites Cu/Ni/TiO_2_/MWCNTs synthesized through hydrothermal methods, which was characterized by various characterization analyses such as XRD, Raman, FESEM, XPS, UV–Visible, and CV technique. The as-prepared Cu/Ni/TiO_2_/MWCNTs nanocomposites were involved for their electrochemical and photocatalytic ability towards FLT drugs. The electrochemical analysis indicates that the synthesized nanocomposites exhibit an excellent electrical activity towards the reduction of FLT with low LOD (0.09492), high sensitivity (1.9288 μA μM^−1^ cm^−2^), with a wide linear range from 10 to 150 μM, and good selectivity. The potential applications of FLT towards the analysis of the real sample were inspected in tap and river water with accessible results obtained. In addition, the Cu/Ni/TiO_2_/MWCNTs nanocomposites exhibit an excellent photocatalytic ability for the degradation of FLT under irradiation of visible light with a higher degradation rate of 75% within 45 min of irradiation time. Moreover, the GC–MS analysis confirmed that the mineralization of FLT under the visible light irradiations. As a result, the synthesized nanocomposites have an excellent electrical and photocatalytic ability. Therefore, nanocomposites have been used for environmental remediation applications.

## Methods

### Synthesis of Cu/Ni/TiO_2_/MWCNTs nanocomposites

Hydrothermal techniques are used to synthesize the nickel/copper doped TiO_2_ nanoparticles loaded into MWCNT. Cu: Ni: TiO_2_ nanoparticles are made using a 1:1:2 of Cu: Ni: TiO_2_, respectively. In the first step, 0.2 mol of titanium tetra isopropoxide is added dropwise to 30 ml of absolute ethanol and stirred continuously for 1 h. Subsequently, 0.1 mol of copper nitrate and nickel nitrate is dissolved in 20 ml of absolute ethanol. The copper and nickel solutions are then added dropwise to the TiO_2_ solution and stirred uniformly for 1 h. Thereafter, 0.1 mol of NaOH solution is added to the above solution and the pH of the solution is maintained at 7. Therefore, the highly homogenous sol–gel is obtained after 1-h magnetic stirring. A 100 mg of MWCNT is dispersed to the above solution with constant stirring. The prepared solution is further transferred into a Teflon-lined stainless-steel autoclave, which is placed in a hot air oven at 120 °C for 12 h. When the hydrothermal process is over, the autoclave is cooled naturally. In addition, the solution is washed with DI water and absolute ethanol multiple times to eliminate any other residues and impurities. The precipitate is then dried at 90 °C in a hot air oven overnight to evaporate excess water molecules and organic residues. Finally, the dried powder is calcinated at 600 °C for 3 h to obtain nanoparticles.

### Characterization of prepared nanocomposites

As prepared Cu/Ni/TiO_2_/MWCNTs nanocomposites were studied using various analytical techniques. The crystalline structure and crystalline size were studied using X-ray diffraction (XRD, D2 Phaser, Bruker) with monochromatic CuKα radiation (λ = 1.540 Å). The synthesized Cu/Ni/TiO_2_/MWCNTs nanocomposites surface morphology and topography are analyzed by field-emission scanning electron microscope (FESEM-EDX, JEOL, JSM-7610F, and Hitachi Regulus 8100). In addition, the prepared nanocomposite's chemical composition is verified by X-ray photoelectron spectroscopy (XPS) (Thermo Scientific Multilab 2000 XPS). The nanocomposite modified electrode activities, which means electrochemical sensor performance is studied by cyclic voltammetry (CV) and differential voltammetry (DPV). A CHI 211B electrochemical workstations (CH Instruments Co., Austin, TX, USA) are used to measure all the electrochemical experiments. A three-electrode device, with a reference electrode, counter electrode, and working electrode, is used for voltammetry studies. The reference, counter, and working electrodes are made up of Ag/AgCl, platinum wire, and SPCE, respectively. For electrochemical studies are performed at room temperature, the Suntex pH meter is used to determine the pH. Moreover, the photocatalytic degradation of the FLT aqueous solution was analyzed by using a UV–Visible spectrometer.

### Photocatalytic degradation of Furaltadone

The copper and nickel-doped TiO_2_ nanoparticles are loaded multiwall carbon nanotubes (MWCNTs) used as a visible-light-active photocatalysis. The synthesized nanocomposite photocatalytic performance is studied by degradation of furaltadone organic compounds under visible light irradiation. The tungsten-halogen lamp (500 W, 110 V) is used as a visible light source. For the treatment process, 36 mg of model pollutant is mixed into 100 ml of DI water, which is stirred magnetically for a few minutes under dark conditions to achieve homogeneous mixtures. The degradation process is carried out with different photocatalysis concentrations. Before the degradation process, the calculated amount of synthesized photocatalysis is dispersed in a furaltadone aqueous solution with magnetic stirring for absorption and adsorption of photocatalysis into organic molecules. A 100 ml aqueous solution of furaltadone is used for each experiment. The furaltadone aqueous solution is approximately 20 cm away from the visible light source. During the treatment process, a water circulation unit is used to maintain the solution temperature at room temperature. The same procedure is used for each photocatalysis treatment condition. The treatment process is carried out by various treatment times from 0 to 45 min. A 5 ml of the aqueous solution is drawn every 5 min for degradation studies. The aqueous solution is filtered with Whatman 40 filter paper to avoid interferences. Finally, a UV–Visible absorption spectrometer is used to characterize the filtered aqueous solution for the concentration of furaltadone at their characteristic wavelength of 360 nm. The degradation and decolorizations value of furaltadone aqueous solution is examined by the following expression^15^.

$${\text{Degradation}}\;{\text{Efficiency = }}\frac{{C_{0} - C_{t} }}{{C_{0} }} \times 100\%$$

where *C*_0_ and *C*_*t*_ are denoted as before and after treatment of furaltadone concentration, respectively.

## Supplementary Information

Supplementary Information 1.

Supplementary Information 2.

Supplementary Information 3.

Supplementary Information 4.
