# Supplementary figures and images for: Investigation of electrocatalytic and photocatalytic ability of Cu/Ni/TiO2/MWCNTs Nanocomposites for detection and degradation of antibiotic drug Furaltadone
Source: Sci Rep. 2022 Jan 18;12:886. doi: 10.1038/s41598-022-04890-z (PMC8766570; doi:10.1038/s41598-022-04890-z)

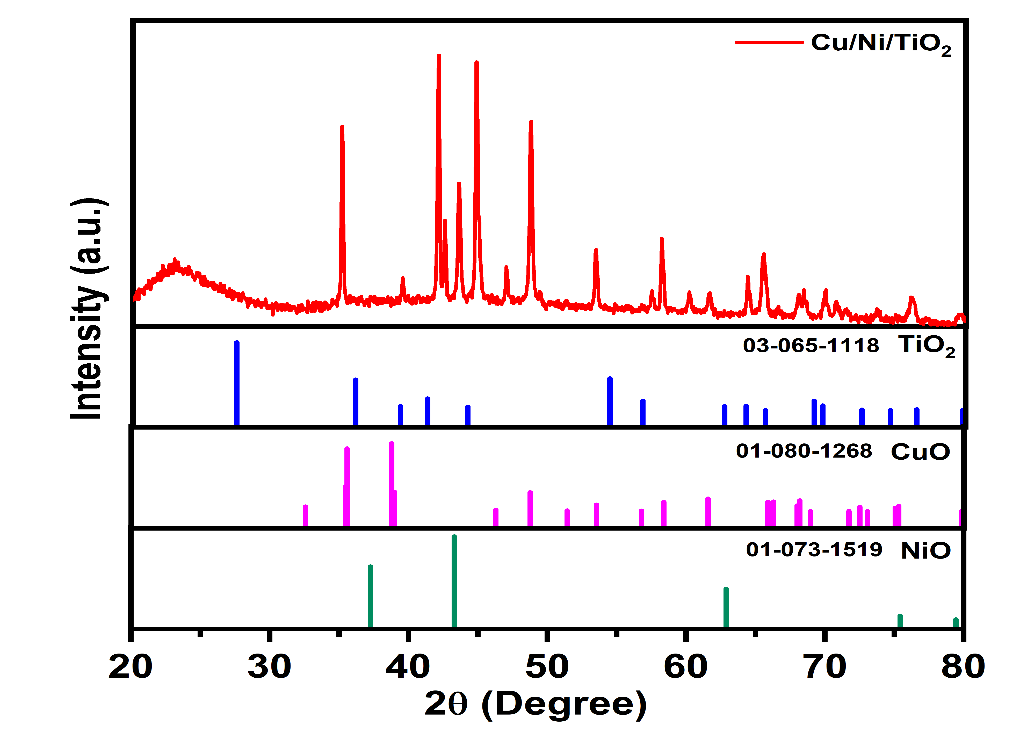

Supplement: Supplementary file 1 — Supplementary Information 1. [file 41598_2022_4890_MOESM1_ESM.tif]

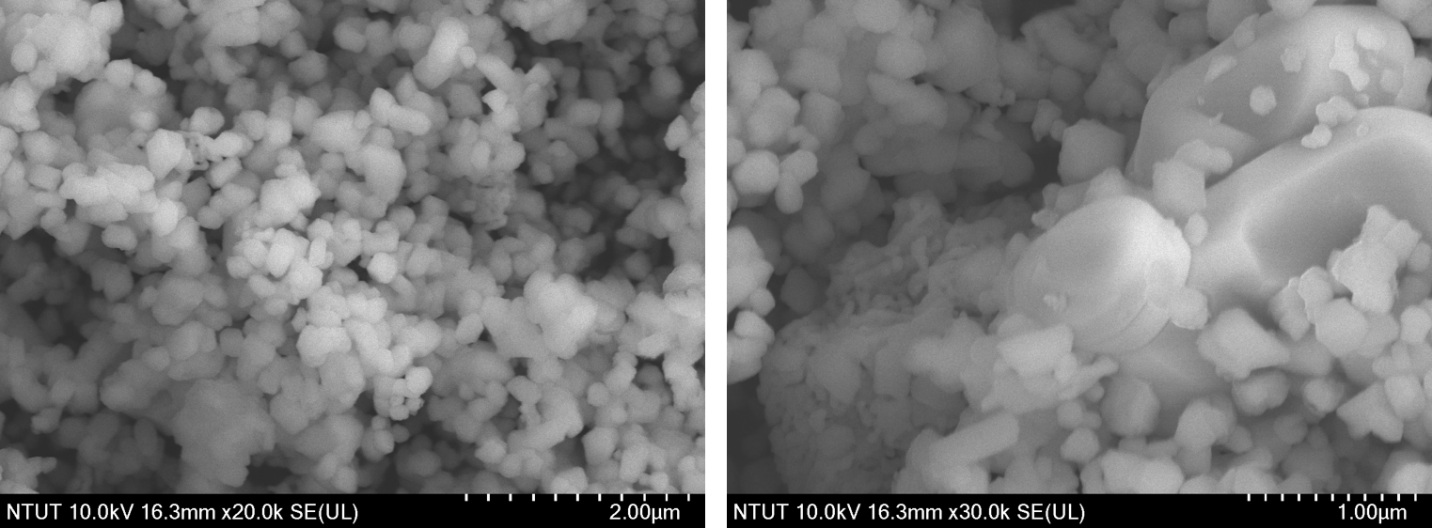

Supplement: Supplementary file 2 — Supplementary Information 2. [file 41598_2022_4890_MOESM2_ESM.tif]

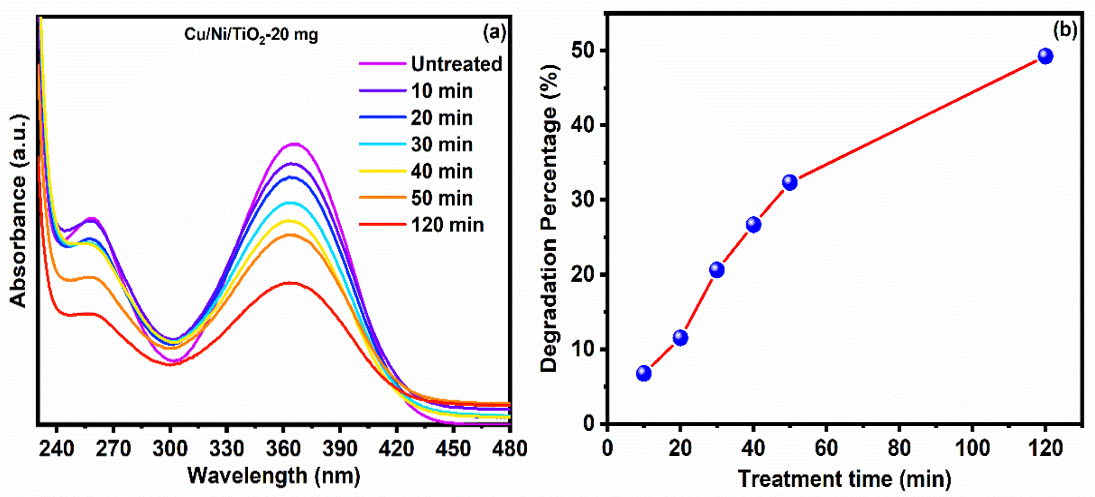

Supplement: Supplementary file 3 — Supplementary Information 3. [file 41598_2022_4890_MOESM3_ESM.tif]
