# Supplementary material for: Investigation of electrocatalytic and photocatalytic ability of Cu/Ni/TiO2/MWCNTs Nanocomposites for detection and degradation of antibiotic drug Furaltadone
Source: Sci Rep. 2022 Jan 18;12:886. doi: 10.1038/s41598-022-04890-z (PMC8766570; doi:10.1038/s41598-022-04890-z)
Supplement: Supplementary file 4 — Supplementary Information 4. [file 41598_2022_4890_MOESM4_ESM.docx]

**Supplementary Materials**

**Investigation of electrocatalytic and photocatalytic ability of Cu/Ni/TiO_2_/MWCNTs Nanocomposites for detection and degradation of antibiotic drug Furaltadone**

Dhanapal Vasu, Arjunan Karthi Keyan, Subramanian Sakthinathan^*^, Te-Wei Chiu^*^,

^a^ Department of Materials and Mineral Resources Engineering, National Taipei University of Technology, No. 1, Section 3, Chung-Hsiao East Road, Taipei 106, Taiwan, ROC.

** Corresponding author: E-mail address:* [*sakthinathan1988@gmail.com*](mailto:sakthinathan1988@gmail.com)*,* [*tewei@ntut.edu.tw*](mailto:tewei@ntut.edu.tw)


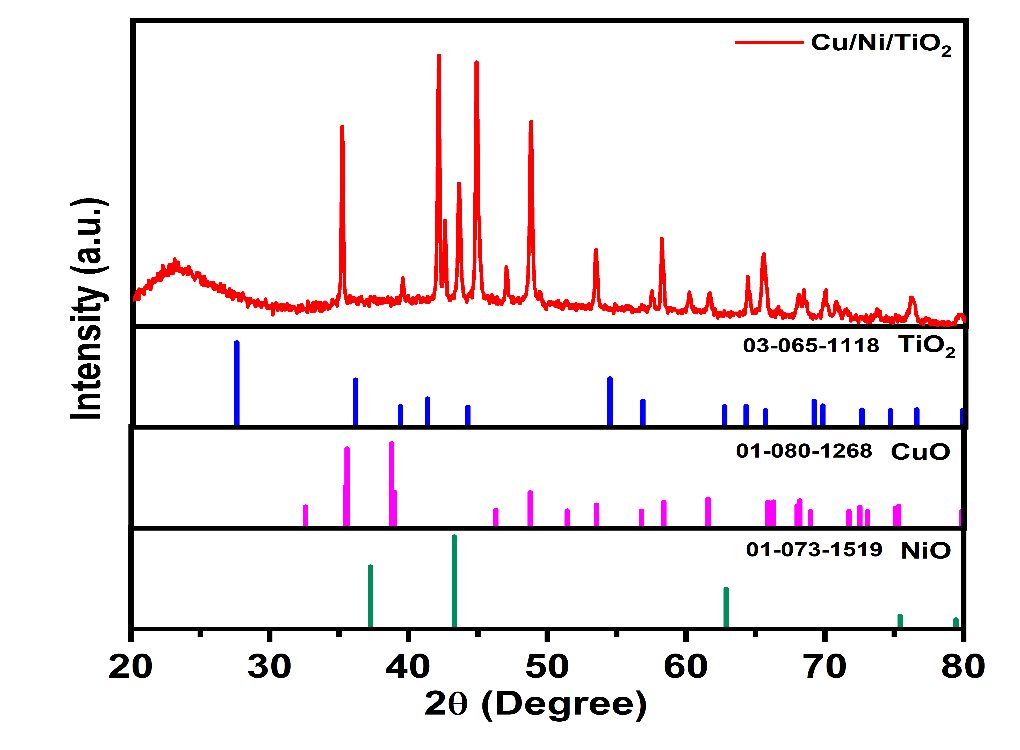


**Figure S1.** **XRD patterns of Cu/Ni/TiO_2_ nanocomposites.**


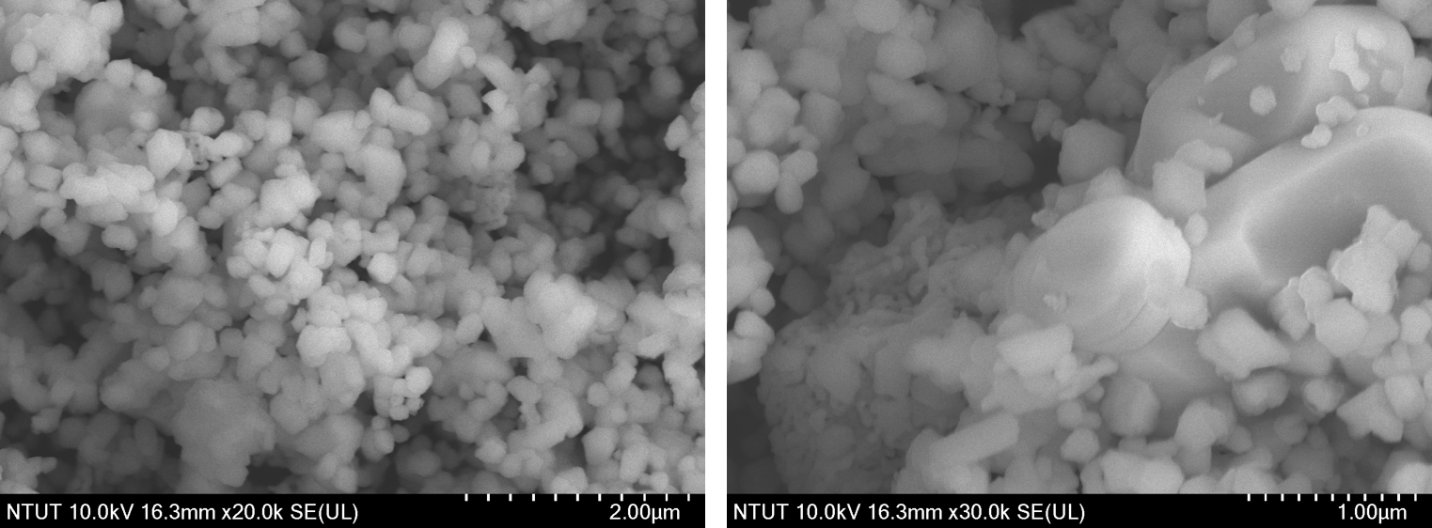


**Figure S2.** **The synthesized Cu/Ni/TiO_2_ nanocomposites FESEM analysis.**


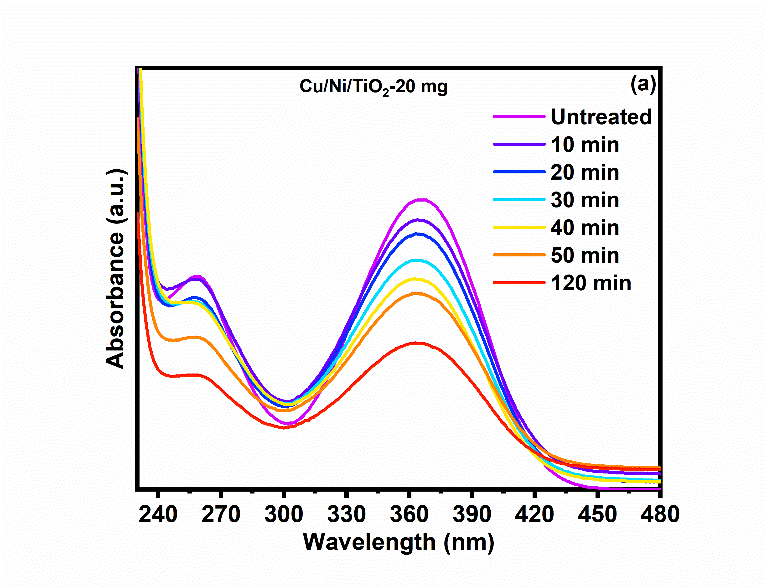

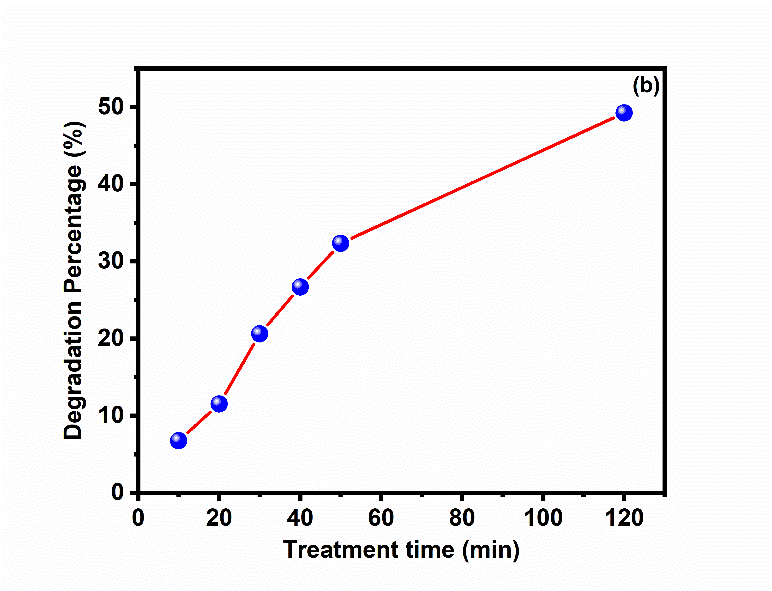


**Figure S3.** **(a)** **Degradation of FLT using Cu/Ni/TiO_2_ nanocomposites, and (b) FLT degradation percentage**
